# Interactions between symptoms and psychological status in irritable bowel syndrome: An exploratory study of the impact of a probiotic combination

**DOI:** 10.1111/nmo.14477

**Published:** 2022-09-30

**Authors:** David Groeger, Eileen F. Murphy, Hern Tze Tina Tan, Ida Søgaard Larsen, Ian O'Neill, Eamonn M. M. Quigley

**Affiliations:** ^1^ Precisionbiotics Group Cork Ireland; ^2^ Novozymes A/S Bagsværd Denmark; ^3^ Department of Microbiology, APC Microbiome Ireland National University of Ireland Cork Ireland; ^4^ Division of Gastroenterology and Hepatology, Lynda K and David M Underwood Center for Digestive Disorders, Houston Methodist Hospital Weill Cornell Medical College Houston Texas USA

**Keywords:** anxiety, cortisol, depression, inflammation, irritable bowel syndrome, sleep, stress

## Abstract

**Background:**

Stress is an exacerbator of irritable bowel syndrome (IBS) symptoms, and anxiety and depression are co‐morbidities. *Bifidobacterium longum* strains 1714® and 35642® attenuate stress responses in healthy people and reduce symptoms in IBS, respectively. Here, we explore relationships between the psychological and visceral effects of the two strains (COMBO) in IBS subjects and biomarkers of stress and inflammation.

**Methods:**

We recruited 40 patients with IBS (Rome III) and mild to moderate anxiety (HADS‐A) and/or depression (HADS‐D) and 57 asymptomatic female controls with low or moderate stress. IBS patients were fed COMBO (1 × 10^9^ cfu/day) for 8 weeks with an 8‐week washout. IBS symptoms, psychometric measures, salivary cortisol awakening response (CAR), and plasma inflammatory biomarkers were assessed every 4 weeks.

**Key results:**

Compared to healthy controls, IBS subjects had a blunted CAR. Treatment with COMBO restored CAR and improved IBS symptoms compared to baseline during the treatment phase. The COMBO reduced HADS‐D, HADS‐A score, and TNF‐α, while sleep quality improved significantly from baseline to the end of the intervention. Surprisingly, these parameters improved further once treatment ended and maintained this improvement by Week 16.

**Conclusions and inferences:**

These findings suggest that the stress response is a major driver of IBS symptoms. The time course of the beneficial effect of COMBO on IBS symptoms suggests that this is achieved through a restoration of the stress response. In contrast, the time course of the effects of COMBO on anxiety and depression in IBS paralleled an anti‐inflammatory effect as indicated by a reduction in circulating levels of TNF‐α.

## Graphical abstract

The probiotic combination restored the blunted cortisol response associated with IBS which was mirrored by clinically significant improvements in overall IBS symptoms, but the effect lasted only for the duration of treatment. The probiotic combination reduced depression, anxiety scores, and circulating levels of TNFα, as well as improving sleep quality.

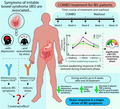

**Key points**

- Irritable bowel syndrome (IBS) is a disorder of gut‐brain interaction and has been linked, in the gut, to a disturbed microbiome and an altered immune response and, in the brain, to sensitivity to stress and co‐morbid anxiety and depression.
- Certain probiotics have been shown to impact on symptoms thought to originate in either the gut or the central nervous system.
- By studying the response, in IBS, to a combination of a centrally and a peripherally acting probiotic, we were able to differentiate between impacts on stress‐related effects on gut symptoms (mediated by cortisol) and on anxiety and depression (mediated by pro‐inflammatory cytokines).

## 1 INTRODUCTION

Irritable bowel syndrome (IBS) is a common, typically chronic, disorder whose central features are recurrent episodes of abdominal pain and disturbed bowel habit.^1^, ^2^ Though non‐fatal, IBS impairs quality of life (QOL) and inflicts significant burdens on sufferers and healthcare systems.^1^ The underlying factors contributing to IBS are heterogeneous and not completely understood but it is considered that dysmotility, visceral hypersensitivity, immune activation, brain–gut interactions, and changes in gut microbiota are significant contributors to its pathophysiology.^1^, ^3^, ^4^, ^5^

Stress is an exacerbator of IBS symptoms, and anxiety and depression are common co‐morbidities.^4^, ^6^, ^7^, ^8^, ^9^ When present, anxiety and depression exacerbate IBS symptoms, impair QOL, and promote healthcare‐seeking behaviors; thus, resulting in increased healthcare utilization and costs.^1^, ^10^ IBS patients reporting psychological distress demonstrated more severe GI and non‐GI symptoms, fatigue, GI‐specific anxiety, and lower QOL as well as impaired response to therapy.^11^, ^12^, ^13^, ^14^ Furthermore, both anti‐depressants^15^ and psychological interventions have been shown to be efficacious in reducing symptoms in IBS.^16^, ^17^

Consequently, therapies that address gastrointestinal symptoms alone may fail to alleviate burdens on daily life and activities. For example, we have previously shown that *Bifidobacterium longum* 35642® provides relief of global as well as individual cardinal symptoms in IBS but did not impact on QOL or anxiety and depression.^18^ Another *B. longum* strain, *B. longum* 1714®, in contrast, has been shown to attenuate stress responses in healthy adults.^19^ We hypothesized that a combination of the two strains would provide a further opportunity to provide insights into relationships between visceral and psychological symptoms and biological parameters in IBS. However, the two strains have not been tested in combination, and the mechanisms of their combined effects are unknown. Here we, therefore, studied the psychological and visceral effects of the two strains in combination (COMBO) in IBS subjects and explored associations with a variety of symptoms and biomarkers of stress and inflammation.

## 2 MATERIALS AND METHODS

### 2.1 Participants with IBS

We recruited 40 female adult patients (ages 18–65 years) with IBS and mild to moderate anxiety and/or depression using the Hospital Anxiety and Depression scale (HADS; HADS‐A or HADS‐D scores ranging from 8 to 14). The Rome III criteria were used to diagnosis IBS and required that over the preceding 3 months subjects had to have abdominal discomfort or pain more than 25% of the days that was associated with two of three features: (1) relieved with defecation, (2) onset associated with a change in frequency of stool, or (3) onset associated with a change in form (appearance) of stool.

Patients with a significant acute or chronic coexisting illness, major inflammatory disorders, severe immunodeficiency, abdominal surgery (except for hernia repair and appendectomy), lactose intolerance, history of psychiatric illness other than anxiety or depression, were excluded. Patients who were pregnant/breast‐feeding, had a body mass index (kg/m^2^) >30 or taking anti‐depressants, anxiolytics, antipsychotics in the last 6 months were also excluded. The probiotic Alflorex® with the *B. longum* 35624® strain was prohibited for 6 months while all other probiotics were forbidden for 4 weeks prior to and during the study. Antibiotics were forbidden for 3 months prior to the commencement of the study. Patients were instructed to follow their habitual diet and exercise routine and not consume any disallowed medications, supplements, probiotic products, or dietary fiber, that could interfere with the assessment of the study product for the duration of the study. The use of rescue medication (bisacodyl 5 mg for constipation if no bowel movement occurred on four consecutive days or loperamide 2 mg for troublesome diarrhea) was recorded. Each potentially eligible patient was evaluated by a full review of clinical history, physical examination, full blood count, and routine biochemistry analysis. Subjects with any clinically significant abnormalities in any of these tests were excluded from the study.

### 2.2 Low and moderate stress non‐IBS controls

Two groups of healthy female non‐IBS controls were recruited to provide a sex‐matched comparison with the IBS patient cohort. Participants were categorized into either moderate stress group based on a Cohen's Perceived Stress Scale (PSS) score^20^ of 14–26 (moderate) and HADS‐A OR HADS‐D of 8–14 or into a low stress group based on a PSS‐10 score of ≤12, and HADS‐A and HADS‐D scores ≤7. Participants with a history of functional GI disorders as defined by ROME III and serious health conditions (as described above) were excluded. Healthy controls were instructed to follow their habitual diet and exercise routine and not consume any disallowed medications, supplements, probiotic products, or dietary fiber, that could interfere with the assessment of the baseline characteristics. Subjects who were taking psychoactive medications at the time of the study or within the last month were also excluded from the study. The low and moderate stress (non‐IBS control subjects) were assessed at baseline and acted as comparators to the IBS patients in terms of their psychometric, HPA, and inflammatory activity. They were studied at baseline only and were not followed for 16 weeks.

### 2.3 Design of the intervention study in IBS subjects

**FIGURE 1:**
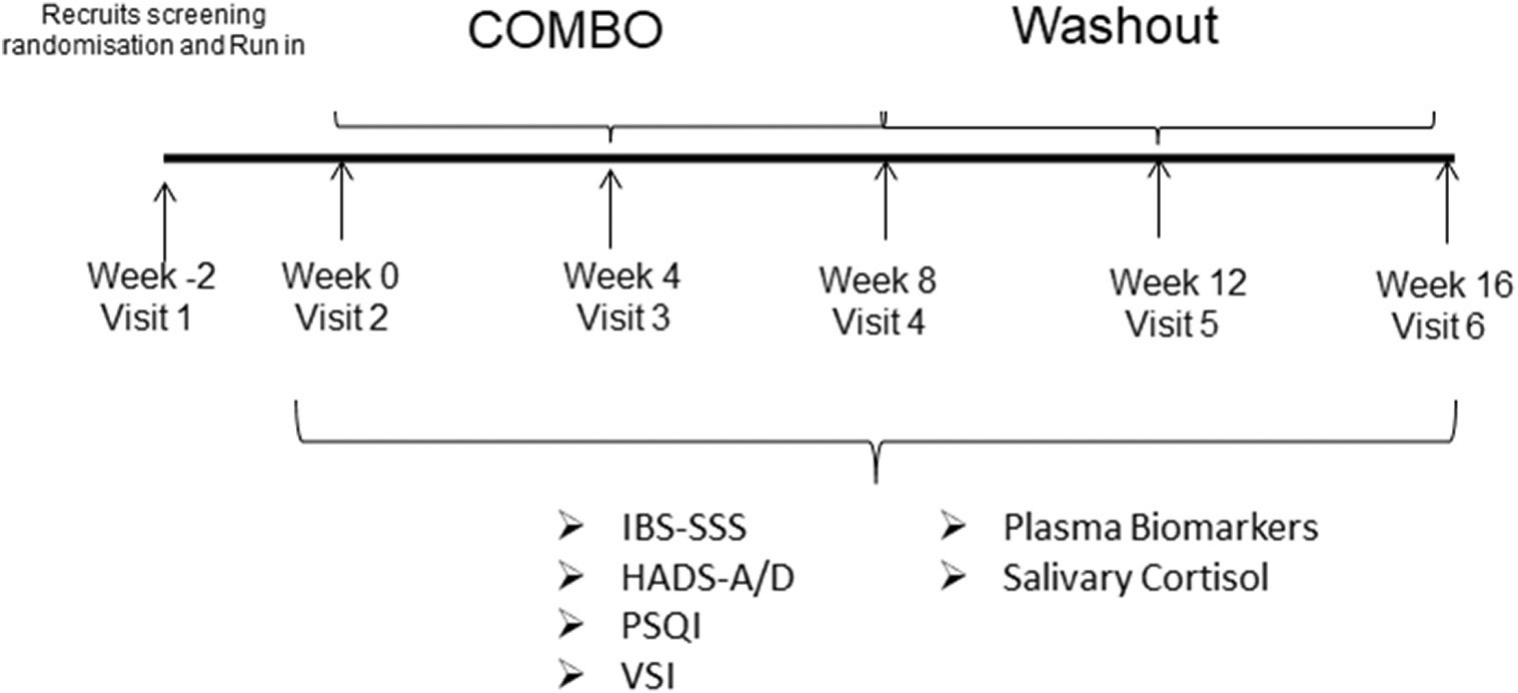
Schematic of the study design

As this was an exploratory study, the trial was not powered, and 40 IBS subjects were selected for the study. Data from this study will be used to provide an indication of the effect size and, thereby, power future double‐blind placebo‐controlled studies. All IBS subjects consumed the strain combination “COMBO” product for 8 weeks with an 8‐week follow‐up (Figure 1). The “COMBO” product (*B. longum* 1714® and *B. longum* 35624®) at a dose of 1 × 10^9^ cfu/day total was supplied by Precision Biotics Group. The strain combination was in capsule form and taken once a day. Participants were instructed to consume the product every morning, before, with, or after food. Study visits were conducted at 0, 4, 8, 12, and 16 weeks. Subjects returned any used and unused study product and compliance was assessed at each visit; those subjects who did not comply with product consumption (over 80%) were to be excluded.

### 2.4 Study measures

All self‐reported measures were conducted at 0, 4, 8, 12, and 16 weeks. IBS symptoms were assessed by the IBS Severity Scoring System (IBS‐SSS), which has been validated in outpatients with IBS.^21^ A clinically significant improvement was defined as a reduction in IBS‐SSS by 50 points, as per the original description of the tool. Anxiety and depression were assessed by the HAD scale.^22^, ^23^, ^24^ A clinically significant improvement was defined as reduction in anxiety and/or depression scores of ≥2 points. This was based on the previously established mean clinically important difference for the depression score on the HAD scale of 1.4.^25^, ^26^ The visceral sensitivity index (VSI) was used to measure of the gastrointestinal symptom‐specific anxiety of patients with IBS^27^ while the 19‐item Pittsburgh Sleep Quality Index (PSQI) was used to measure subjective sleep quality, sleep latency, sleep duration, habitual sleep efficiency, sleep disturbances, use of sleeping medication, and daytime dysfunction. A global PSQI score of greater than 5 is used to distinguish poor from good sleepers.^28^

### 2.5 Cortisol awakening response

Saliva samples for the cortisol awakening response (CAR; four samples/morning (0, 30, 45, and 60 min after waking)) were collected at 0, 4, 8, 12, and 16 weeks using Salivette devices and stored at −80°C. Cortisol levels were determined using a commercially available competitive ELISA (Salimetrics LLC) according to manufacturer's instructions. Cortisol data were excluded if subjects collected their samples more the 5 min outside of the designated collection times.

### 2.6 Cytokine and biomarker assays

Whole blood samples of volume 9 ml were collected at 0, 4, 8, 12, and 16 weeks in tubes containing ethylene diamine tetra acetic acid. Samples were centrifuged immediately and plasma frozen at −80°C. Measurements of plasma TNF‐α, IL‐6, IFN‐α, CRP, and BDNF were performed using an electro‐chemiluminescence multiplex system Sector 2400 imager from Meso Scale Discovery using S‐plex kits measuring from fg/ml to ng/ml.

### 2.7 Safety

Blood samples for blood count, serum chemistry, and quantitative immunoglobulin levels were obtained at initial evaluation and at the end of the study and analyzed using standard laboratory methods to assess patient safety. Adverse and serious adverse events were recorded.

### 2.8 Statistics

All statistical analyses unless stated otherwise were performed using GraphPad Prism for Windows (Version 9.2). Before analysis, the data were examined for normality using the Kolmogorov–Smirnov test, and for homogeneity of variance. Statistical significance for data with normal distribution was evaluated using a one‐way analysis of variance (ANOVA) with Tukey post‐tests, whereas data not normally distributed were analyzed using the Kruskal–Wallis test with Dunn's multiple comparison test. For correlation analysis, simple linear regression was performed where the *R* ^2^ and *p* value were recorded. For CAR, area under the response curve was calculated with respect to increase (AUCi) using the trapezoidal method as an indicator for the physiological stress response.^29^ When comparing the intervention on all biological and symptom measures over time, a mixed effect model was used with Dunnett's post‐test to cope with missing values from protocol violators. Chi‐squared tests were used to determine the interaction between IBS severity and treatment throughout the course of the study. Principal component analysis (PCA) was performed to identify trends in biomarkers or subjective measures at baseline where HADS Depression was classified as low: 1–7, medium: 7–10, and high: >10 and IBS was separated into mild (0–175), moderate (176–300), and severe (301–500) based on the IBS‐SSS scale. Each factor was normalized for value‐mean/standard deviation. PCA analysis with missing values was performed using the following packages “missMDA” and “FactoMineR” and “ggbiplot” used to draw plots in R.^30^ For a post hoc analysis, we categorized our IBS patients into those who had a reduction in depression scores of ≥2 points on HAD scale and those who did not. Permutational multivariate analysis of variance (PERMANOVA) using the Adonis function in the R Vegan package was used to determine significance in dissimilarity matrices across samples by metadata categories (e.g., biomarkers, disease severity, and subjective questionnaires). A heatmap of all normalized data with clusters by sample and biomarker was generated using GraphPad Prism. Change from baseline comparisons were analyzed using one‐way ANCOVA. Unless otherwise indicated, all results were presented as means ± SEM. Differences were considered significant at *p* < 0.05.

## 3 RESULTS

### 3.1 IBS patients baseline characteristics

**FIGURE 2:**
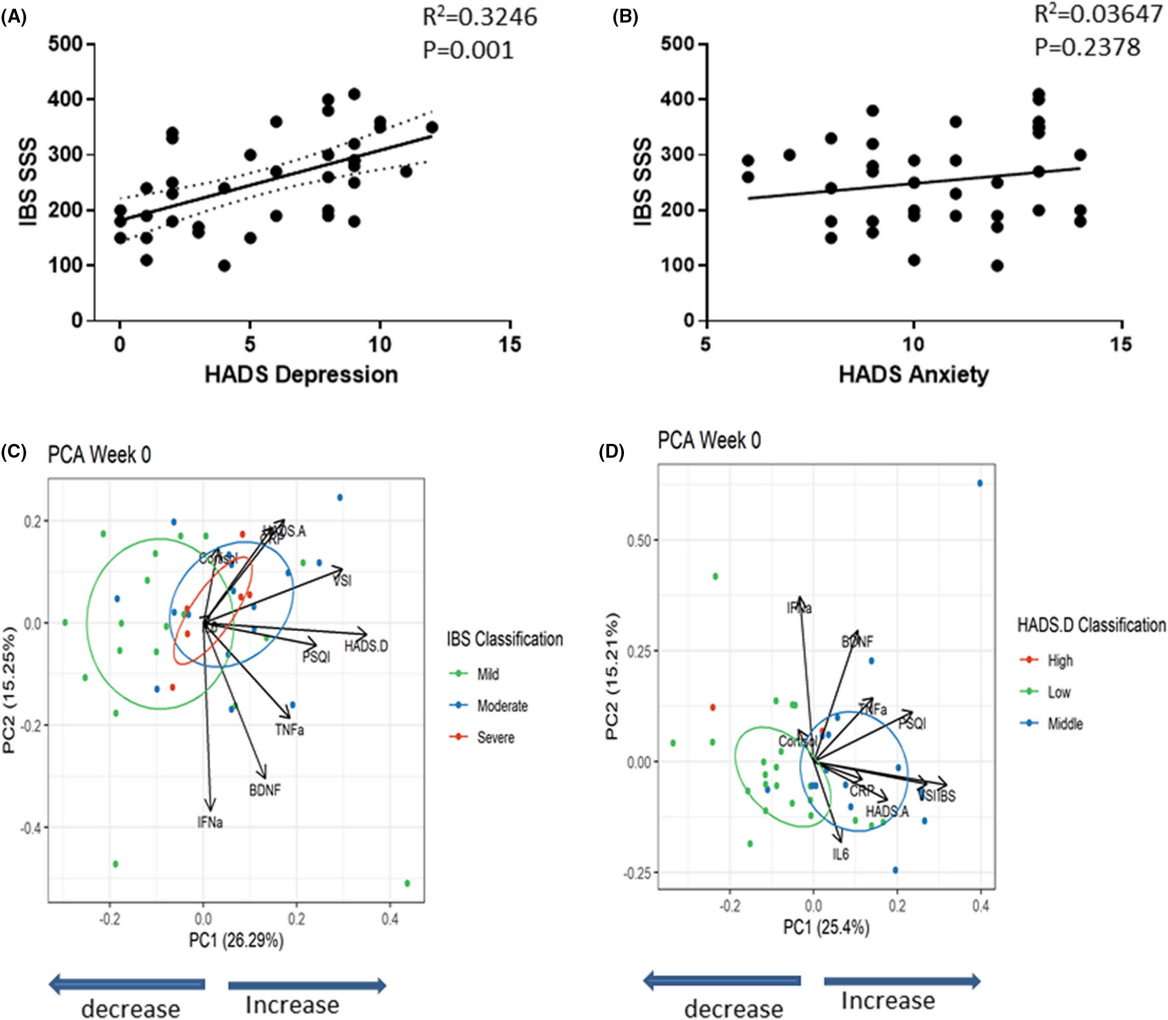
Baseline characteristics: IBS‐SSS is weakly correlated with HADS‐Depression: The degree of HADS Depression is more of a determining factor than GI symptom severity in separating subjects according to their baseline levels of biological and subjective questionaries endpoints. (A) Linear regression analysis between IBS‐SSS and HADS depression (*R* ^2^ = 0.3246 *p* = 0.001). (B) Linear regression analysis between IBS‐SSS and HADS‐Anxiety (*R* ^2^ = 0.03647 *p* = 0.2378). (C) PCA for all biological endpoints and subjective questionnaires in the IBS patients at Week 0, showing no distinct segregation of patients from mild to moderate and severe GI symptoms (IBS‐SSS). Ellipses = 50% CI of the groups. (D) PCA for all biological endpoints and subjective questionnaires in the IBS patients at Week 0, showing distinct segregation of patients from low depression to mid and high HADS (Low: 1–7, Medium: 7–10, High: >10 depression scores). Ellipses = 50% CI of the groups. Too few datapoints in High group to compute CI

A total sample of 40 women with IBS was enrolled into the study and all 40 completed the study with no dropouts. At baseline, their median age was 40 and median IBS‐SSS score 250 with over 82% of subjects in the moderate to severe IBS category (IBS‐SSS > 175). According to the Rome III criteria, IBS patients were designated as having IBS with diarrhea (IBS‐D, *n* = 10), IBS with constipation (IBS‐C, *n* = 13), or mixed IBS (IBS‐M, *n* = 17). Participants predominately suffered from subthreshold anxiety (95%) and to a lesser extent depression (48%) with 38% suffering from both subthreshold anxiety and depression (Table 1). Correlation of HADS‐Anxiety (HADS‐A) and HADS‐Depression (HADS‐D) scores to IBS severity scale (IBS‐SSS) showed that HADS‐D (*R* ^2^ = 0.325; *p* = 0.001; Figure 2A) but not HADS‐A (*R* ^2^ = 0.036; *p* = 0.239) correlated with IBS‐SSS (Figure 2B).

**TABLE 1:** Subject characteristics at the baseline

Demographic characteristics	Low stress	Moderate stress	IBS
Numbers	29	28	40
Age, median (IQR)	47 (39–51.5)	43 (33.25–54.5)	40 (26.25–46)
Sex	Female	Female	Female
IBS‐SSS median (IQR)	–	–	250 (190–315)
IBS‐SSS severe (≥300) [*n* (%)]	–	–	10 (25%)
IBS‐SSS moderate (175–300) [*n* (%)]	–	–	23 (58%)
IBS‐SSS mild (75–175) [*n* (%)]	–	–	7 (18%)
IBS subtype [*n* (%)]			
IBS‐mixed	–	–	17 (42.5%)
IBS‐constipation predominant	–	–	13 (32.5%)
IBS‐diarrhea predominant	–	–	10 (25%)
HADS s [*n* (%)]			
Anxiety (HAD‐A ≥8)	0	27(96%)	38 (95%)
Depression (HAD‐D ≥8)	0	11 (39%)	18 (45%)
Anxiety and depression	0	11(39%)	15 (38%)
PSQI global sleep score [*n* (%)]			
PSQI global score >5	1 (3%)	20 (71%)	26 (65%)

### 3.2 Determinants of IBS symptom severity at baseline

To identify the main contributors to IBS symptoms, normalized biomarker data, extra GI subjective questionnaires (HADS‐A/D, PSQI, and VSI) were subjected to PCA at Week 0 with patients categorized by IBS symptom severity or HADS‐D severity. Interestingly, no separation in the groups in terms of their IBS severity of mild, moderate, and severe was observed (PERMANOVA *p* = 0.25 and *R* ^2^ = 0.061). IBS severity classification does not significantly drive differences in the remaining factors in the analysis (Figure 2C). However, a separation was observed between low and moderate/high HADS‐D patients (PERMANOVA *p* = 0.003 and *R* ^2^ = 0.131). HADS.D severity significantly drives the other included parameters, especially plasma biomarkers (Figure 2D).

### 3.3 Non‐IBS stress controls and IBS sufferers demonstrate similar patterns of subthreshold anxiety and depression

**FIGURE 3:**
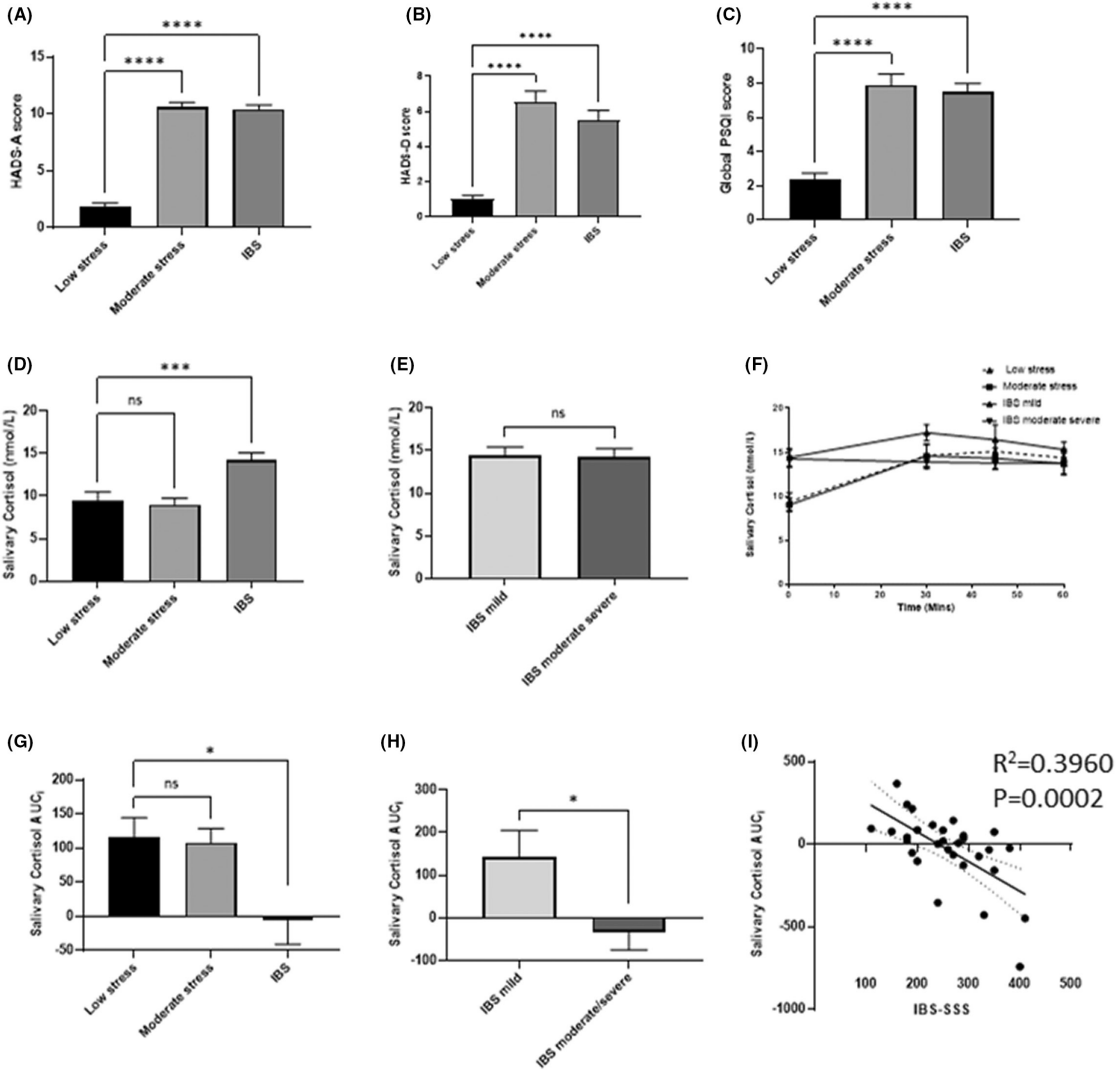
Baseline Characteristics: Psychometric Data: Extra Gastrointestinal Symptoms are similar between moderate stress and IBS patients whereas CAR is blunted in IBS patients in this study and is negatively correlated with IBS‐SSS score (A) HADS Anxiety score was determined at baseline. Data shown are mean ± SEM. ****p < 0.0001 (one‐way ANOVA comparison of moderate stress, IBS vs. low stress group). (B) PSQI global score was determined at baseline. Data shown are mean ± SEM. ****p < 0.0001 (one‐way ANOVA comparison of moderate stress, IBS vs. low stress group). (C) HADS Depression score was determined at baseline. Data shown are mean ± SEM. ****p < 0.0001 (one‐way ANOVA comparison of moderate stress, IBS vs. low stress group). (D) Salivary CAR at baseline‐comparison of low stress to moderate stress and IBS. Data shown are mean ± SEM. ***p < 0.001. (E) Salivary CAR at baseline‐comparison between mild to moderately severe IBS. Data shown are mean ± SEM. (F) Salivary CAR comparison of low stress to moderate stress and mild to moderately severe IBS. Data shown are mean ± SEM. ***p < 0.001, **p < 0.01 (one‐way ANOVA comparison of moderate stress, IBS [mild/moderate/severe] versus low stress group). (G) Salivary CAR. Increase in salivary cortisol within 60 min after awakening, represented as area under the curve with respect to the increase (AUCi). Data shown are mean ± SEM. ***p < 0.001, **p < 0.01 (one‐way ANOVA comparison of moderate stress, IBS vs. low stress group). (H) Salivary CAR. Increase in salivary cortisol within 60 min after awakening, represented as area under the curve with respect to the increase (AUCi). Data shown are mean ± SEM (Mann–Whitney *U*‐test/Wilcoxon rank‐sum test mild vs. moderate to severe IBS). (D) Linear regression analysis between IBS‐SSS and HADS‐Depression (*R* ^2^ = 0.3960 *p* = 0.0002)

Fifty‐seven female non‐IBS stress controls were recruited of which 29 were deemed low stress and 28 were moderate stress at baseline. Their median ages were 47 and 43 years, respectively (Table 1). Comparison of non‐IBS controls with low stress and moderate stress showed a significant increase in HADS‐A (*p* < 0.0001), HADS‐D (*p* < 0.0001), and PSQI (*p* < 0.0001) in the moderate stress group compared to the low stress group to a level that was comparable to the IBS subjects (Figure 3A–C). Rates of anxiety and depression between IBS and non‐IBS moderate stress controls were similar at 95% versus 96% for anxiety compared to IBS, 45% versus 39% for depression, and 38% versus 39% for both, respectively (Table 1). In the female control subjects, there were significant correlations between PSS and three other psychometric measures HADS‐Anxiety (*R* ^2^ = 0.8977, *p* = <0.0001), HADS‐Depression (*R* ^2^ = 0.6576, *p* = <0.0001), and the global PSQI score (*R* ^2^ = 0.5447, *p* = <0.0001).

Interestingly, IBS patients in the mild symptom severity category and those with IBS‐D had similar levels of depression to the low stress, non‐IBS controls whereas moderate to severely symptomatic IBS patients (*p* < 0.0001) and those with constipation (*p* < 0.0001) and mixed types (*p* < 0.0001) had significantly higher levels of depression (Figure S1).

### 3.4 Baseline Morning Free Salivary Cortisol Profiles differ between non‐IBS stressed controls and IBS patients with similar levels of subthreshold anxiety and depression

Area under the curve analysis with respect to increase (AUCi) was used to quantify the cortisol response. Both low and moderate stress non‐IBS controls showed a significant increase in free salivary cortisol within the first 30 min after awakening with no difference between the two groups (Figure 3D). In contrast, at the time of awakening IBS patients (*n* = 40) had significantly higher levels of baseline cortisol compared to both non‐IBS stress controls (Figure 3E) but did not demonstrate the expected rise 30 min after awakening. Area under the curve analysis with respect to increase showed that the CAR was significantly lower for IBS patients than for both moderate and low stress non‐IBS controls (*p* = 0.013; Figure 3F). Interestingly, IBS patients with mild symptoms did not have a blunted CAR compared to IBS patients with moderate to severe symptoms (Figure 3G,H). Therefore, subsequent results focus only on the moderate to severe IBS group. Interestingly, linear regression analysis revealed a correlation between AUCi salivary cortisol and IBS‐SSS (*R* ^2^ = 0.3960, *p* = 0.0002; Figure 3I) but not HADs‐A (*R* ^2^ = 0.009076, *p* = 0.5862) or HADs‐D (*R* ^2^ = 0.01732, *p* = 0.4511).

### 3.5 A combination of *B. longum* 35624® and *B. longum* 1714® restores salivary CAR in moderate to severe IBS patients

As previously mentioned, CAR was significantly lower for IBS patients with moderate to severe symptoms compared to moderate stress controls (Figure 3G,H).

**FIGURE 4:**
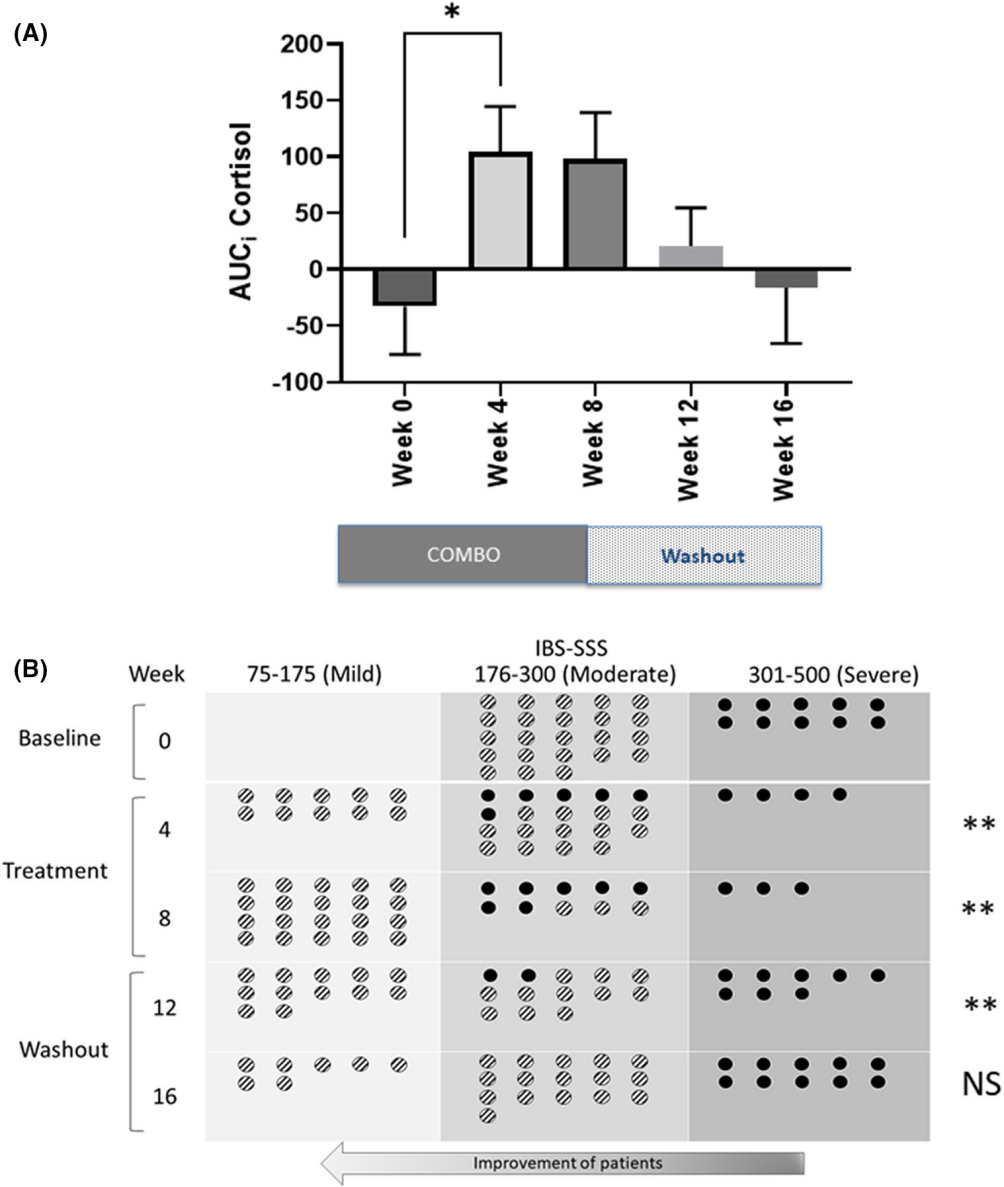
Blunted CAR in moderate to severe IBS patients is normalized after treatment with the COMBO and these patients improve from severe to mild symptoms after treatment with the COMBO. (A) Salivary CAR, increase in saliva cortisol within 60 min after awakening, represented as area under the curve with respect to the increase (AUCi). Data shown are mean ± SEM. *p < 0.05, (one‐way ANOVA comparison of Week 0 to the other time points Weeks 4, 8,12, and 16). (B) Schematic of the improvement of IBS‐SSS symptoms from severe to moderate to mild before and after treatment with the COMBO

Oral administration of the COMBO product to moderate/severe IBS patients significantly increased the CAR (measured as AUC_i_) at Week 4 (*p* = 0.045); *n* = 29 one‐way ANOVA (four saliva collection protocol violators were excluded from the analysis) but not Week 8 (*p* = 0.2081) compared to baseline. Once the treatment of the COMBO product stopped, the CAR returned to baseline levels by Week 8 post‐treatment (Figure 4A).

### 3.6 A combination of *B. longum* 35624® and *B. longum* 1714® decreases symptoms in moderate to severe IBS patients

Shifts from one IBS severity category to another were followed over the duration of the study. As illustrated in Figure 4B, there was significant movement from the severe to moderate and from moderate to mild at 4, 8, and 12 but not at 16 weeks. Chi‐squared tests demonstrated that category of symptom severity and treatment were statistically dependent at Week 4 (*p* < 0.01), Week 8 (*p* < 0.01), and Week 12 (*p* < 0.01); however at Week 16 (8 weeks post‐treatment), there was no significant change in symptom severity category compared to baseline (Figure 4B).

**FIGURE 5:**
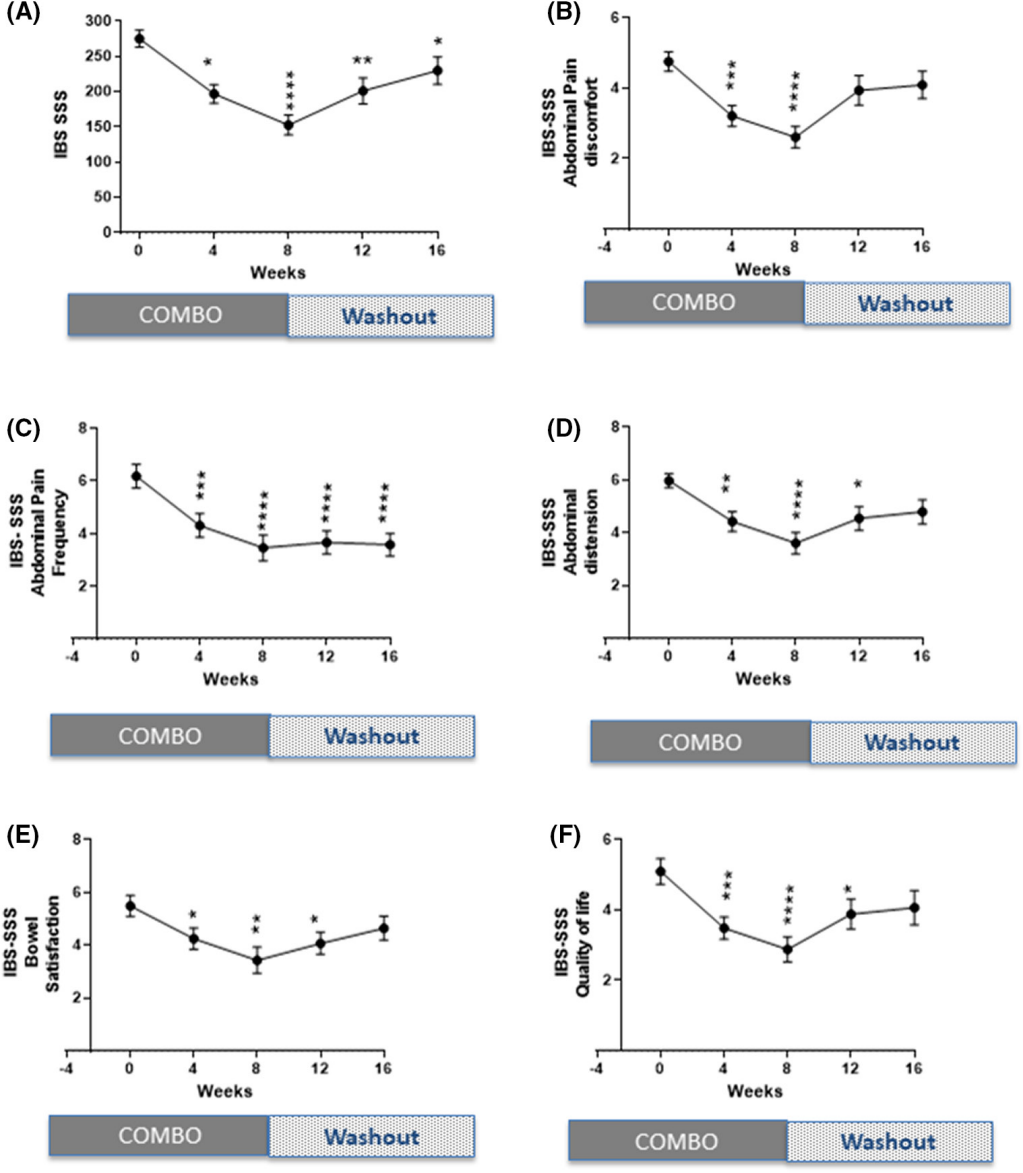
IBS‐SSS symptoms in moderate to severe IBS patients before and after treatment with the COMBO. (A) IBS‐symptom severity score, (B) abdominal pain, (C) abdominal discomfort, (D) abdominal pain frequency, (E) abdominal distension, (F) bowel satisfaction, and (G) IBS QOL. Data shown are mean ± SEM. *p < 0.05 (one‐way ANOVA comparison of Week 0 to the other time points Weeks 4, 8, 12, and 16). IBS‐symptom severity score data shown are mean ± SEM. *p < 0.05 (one‐way ANOVA comparison of Week 0 to the other time points Week 4, 8, 12, and 16). Figures 3A to E are a set of graphs of IBS‐SSS individual symptoms

Compared to baseline, IBS‐SSS scores significantly decreased in response to the COMBO at 4 and 8 weeks (IBS‐SSS 275 ± 68 to 152 ± 79; *p* < 0.0001; *n* = 33 one‐way ANOVA) with the largest effect seen at 8 weeks in the moderate to severe IBS group (Figure 5A). After the 8‐week treatment phase, IBS symptoms slowly increased from Week 8 to Week 16 (IBS‐SSS 152 ± 79 to 229 ± 110) almost returning to baseline scores. A similar response was observed for all the individual component scores of the IBS‐SSS at 4 and 8 weeks: abdominal pain severity (Figure 5B), abdominal pain frequency (Figure 5C), abdominal distension severity (Figure 5D), bowel habit satisfaction (Figure 5E), and QOL (Figure 5F).

Sixty‐six percent and 82% of subjects experienced a clinically meaningful decrease in IBS‐SSS score (≥50) at 4 and 8 weeks, respectively. Following cessation of treatment, IBS‐SSS scores increased with only 40% of the IBS patients showing a decrease in IBS‐SSS ≥50 from baseline to Week 16 (Table S2).

In tandem with the improvement in IBS‐SSS, consumption of COMBO produced a decrease in the daily symptom severity visual analog scales for pain, discomfort, bloating, distension, urgency, straining, and gas by Week 8 (Figure S2). The frequency of bowel movements per day (and type of stools (Bristol stool scale)) was normalized in IBS patients with COMBO treatment (Figure S2).

### 3.7 Effects of *B. longum* 35624® and *B. longum* 1714® on anxiety and depression, sleep quality, and visceral hypersensitivity in moderate to severe IBS patients

The HADS depression score decreased significantly from baseline to the end of the treatment (8 weeks) by a mean ± SD of −1.939 ± 3.152 (*p* < 0.05) in the treatment group. Surprisingly, once treatment stopped the HADS depression score improved further at Week 12 (−2.33 ± 2.63; mean ± SD [*p* < 0.001]) and maintained this improvement by Week 16 (−2.12 ± 3.386; mean ± SD (*p* < 0.0001); 8 weeks post‐treatment; Table 2; Figure S3). A similar response was noted for the HADS anxiety score, with a significant improvement from baseline to the end of the treatment by 8 weeks (mean ± SD of −1.401 ± 2.914 (*p* < 0.05)) a further improvement at Week 12 (−2.333 ± 3.705 (*p* < 0.0001)) and this improvement maintained to Week 16 (−2.394 ± 3.191 (*p* < 0.0001); Table 2; Figure S3). In agreement with the HADS, the VSI showed significant decreases at Weeks 4, 8, 12, and 16 (Table 2; Figure S3). A clinically important difference has been described as an HADS improvement of ≥2. Fifty‐two percent of patients with moderate to severe IBS had an improvement in HADS‐D of 2 or greater after 4 weeks of COMBO consumption, whereas 48%, 55%, and 61% patients were improved at Weeks 8, 12, and 16, respectively (Table S2). We also observed clinical important difference of ≥2 in the HADS‐A score in 36%, 45%, 61%, and 61% of patients at Weeks 4, 8, 12, and 16 (Table S2).

**TABLE 2:** Changes over the duration of the study in plasma biomarkers and measures obtained from subjective questionnaires

Median (IQR) change from baseline (*p* value)					
Test	Week 0	Week 4	Week 8	Week 12	Week 16
Plasma biomarkers					
IL‐6 (fg/ml)	818.9 (632.2–1210)	914.9 (582.7–1305)	1065 (676.6–1436)	905.6 (614.3–1365)	877.1 (606–1287
CRP (mg/L)	1.623 (0.8384–3.809)	1.445 (0.6047–3.312)	1.498 (0.5914–3.230)	–	1.701 (0.6313–5.890)
TNF‐α (fg/ml)	168.7 (147.0–199.9)	164.8 (134.8–197.7)	161.5 (139.3–197.7)	153.8 (122.8–190.0) (*p* < 0.01)	146.2 (127.0–187.5) (*p* < 0.01)
IFN‐α (fg/ml)	83.75 (61.20–124.0)	76.43 (54.09–135.6)	96.45 (64.40–150.1)	117.1 (75.31–176.0)	77.63 (55.01–113.1)
BDNF (pg/ml)	1689 (1317–2614)	2658 (1203–5884) (*p* < 0.05)	2106 (1182–2851)	3347 (1917–5858)	2131 (1048–3759)
Subjective questionaries					
Viseral Sensitivity Index	49.00 (36.00–58.50	44.00 (28.50–49.00) (*p* < 0.01)	41.00 (22.50–52.00) (*p* < 0.01)	42.00 (24.50–54.00) (*p* < 0.01)	42.00 (25.50–54.00) (*p* < 0.05)
HADS‐Depression	8.000 (2.000–9.000)	5.000 (1.000–6.500) (*p* < 0.05)	3.000 (2.000–7.000) (*p* < 0.05)	3.000 (1.000–6.000) (*p* < 0.001)	4.000 (1.000–7.000) (*p* < 0.0001)
HADS‐Anxiety	10.00 (8.75–13.00)	9.000 (6.750–11.00)	8.000 (7.000–11.00) (*p* < 0.05)	8.000 (5.000–11.00) (*p* < 0.0001)	8.000 (6.000–10.00) (*p* < 0.0001)
PSQI global score	7.000 (5.000–10.00)	6.000 (3.000–9.500) (*p* < 0.05)	6.000 (4.000–10.00) (*p* < 0.05)	6.000 (4.000–9.000)	7.000 (3.000–9.000) (*p* < 0.05)

At baseline, the mean PSQI for the IBS patients was 7.67 ± 3.19 with 90% categorized as poor sleepers based on a cutoff of global score at 5 and over. While there was a significant reduction in the PSQI in these IBS patients at Weeks 4, 8, and 16, this reduction was sufficient to move 39% of patients into the good sleep category during the trial (Table 2; Figure S3).

### 3.8 Relationships between HADS scores, sleep quality, VSI, and peripheral proinflammatory cytokines

**FIGURE 6:**
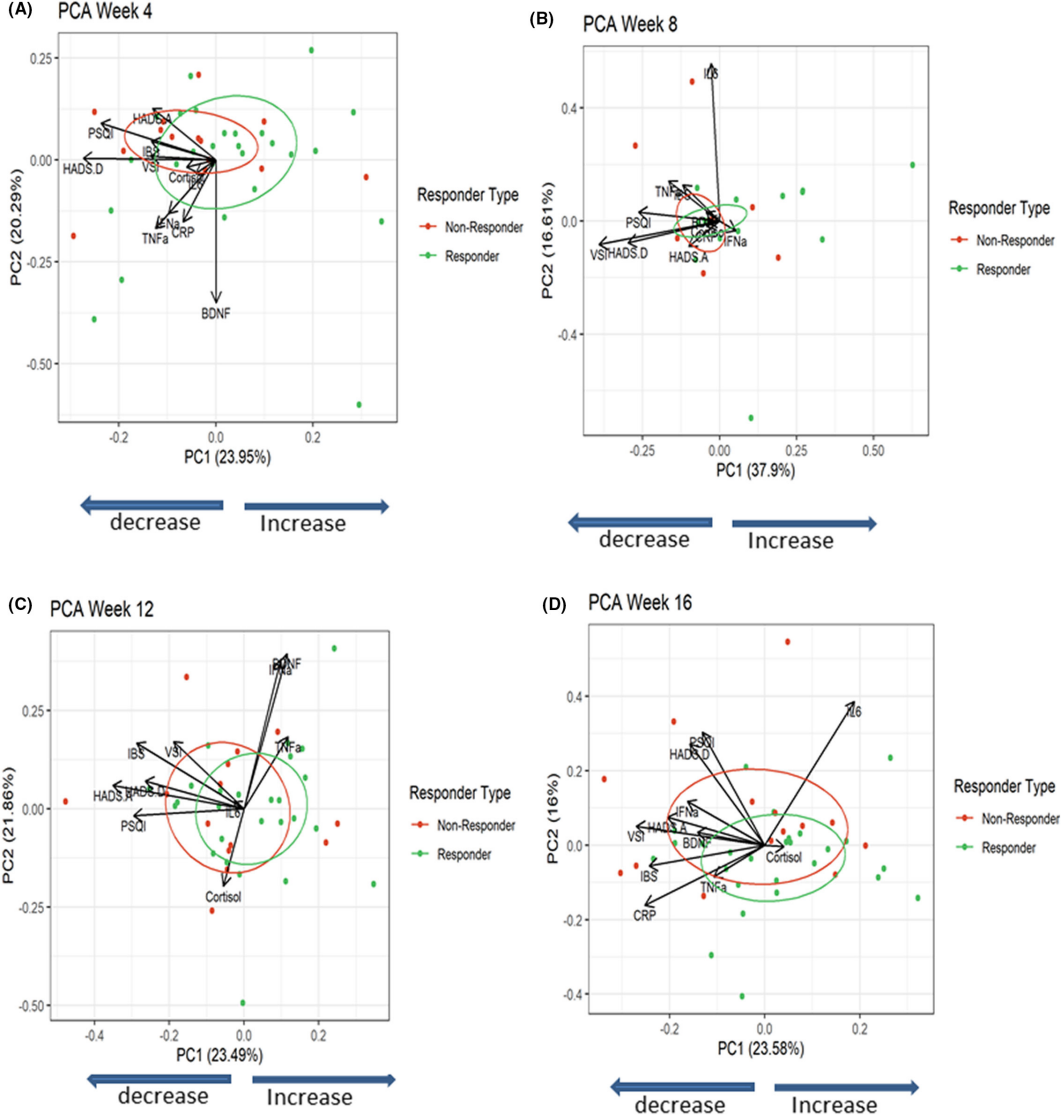
Improvements in HADS Depression are a determining factor in selecting responders and non‐responders to the COMBO. (A) PCA for all biological endpoints and subjective questionnaires in the IBS patients at Week 4, showing distinct segregation of patients who had an improvement ≥2 in HADS depression and those who did not. Ellipses = 50% CI of the groups. (B) PCA for all biological endpoints and subjective questionnaires in the IBS patients at Week 8, showing distinct segregation of patients who had an improvement ≥2 in HADS depression and those who did not. Ellipses = 50% CI of the groups. (C) PCA for all biological endpoints and subjective questionnaires in the IBS patients at Week 12, showing distinct segregation of patients who had an improvement ≥2 in HADS depression and those who did not. Ellipses = 50% CI of the groups. (D) PCA for all biological endpoints and subjective questionnaires in the IBS patients at Week 16, showing distinct segregation of patients who had an improvement ≥2 in HADS depression and those who did not. Ellipses = 50% CI of the groups. CRP data are not included.

There were no baseline differences in the levels of plasma TNF‐α between non‐IBS controls in the low and moderate stress groups or compared to IBS patients with different subtypes and different levels of severity (Table S1; Figure S4). Treatment with COMBO decreased plasma TNF‐α significantly over time in the IBS cohort, an effect that was evident for up to 16 weeks (Table 2; Figure S4). There were no baseline differences in the levels of plasma BDNF between non‐IBS controls in the low and moderate stress groups compared to IBS patients with different subtypes and different levels of severity (Table S1; Figure S4). Treatment with COMBO increased plasma BDNF significantly over time in the IBS cohort and this effect was significant at Week 4 with a trending increase at Week 12 (*p* = 0.07; Table 2; Figure S4). Using PCA to compare IBS patients who had a clinical improvement in HADS‐D of ≥2 in response to the COMBO to those who did not, PCA showed that differences at Weeks 4 (PERMANOVA *p* = 0.14 and *R* ^2^ = 0.038), 8 (PERMANOVA *p* = 0.073 and *R* ^2^ = 0.047), 12 (PERMANOVA *p* = 0.053 and *R* ^2^ = 0.048), and 16 (PERMANOVA *p* = 0.031 and *R* ^2^ = 0.052) were driven by significant decreases in biomarkers TNF‐α, IL‐6, CRP, and subjective measures HADS Anxiety/Depression and PSQI global score (Figures 6A–D and S5; Table S3).

## 4 DISCUSSION

In this study, only IBS subjects with moderate‐to‐severe symptoms demonstrated a blunted CAR compared to non‐IBS stress controls despite similar levels of anxiety, depression, and poor sleep. Furthermore, the COMBO restored the cortisol response and produced parallel and clinically significant reductions in overall and individual IBS symptoms, but this effect lasted only while treatment continued. The COMBO also produced reductions in depression and anxiety scores, as well as the VSI and improved sleep quality. However, in contrast to the IBS response, effects of COMBO on anxiety and depression continued for up to 8 weeks following cessation of treatment and corresponded to reduced circulating levels of peripheral proinflammatory cytokines also evident for up to 16 weeks. This was most notable in subjects who experienced a clinically significant improvement in depression where they had significant decreases in IL‐6, CRP, and tumor necrosis factor alpha (TNF‐α) compared to those who experienced no improvement in depression. These findings suggest that the stress response is a major driver of symptoms in IBS and the clinical response may have been achieved through a restoration of the stress response. The time course on central responses differed and paralleled that of a reduction in circulating levels of the proinflammatory cytokine TNF‐α suggesting that different mechanisms may underpin IBS symptoms and comorbid psychological status.

As prevalence rates of IBS are higher in females than males,^2^ many investigations focus upon female subjects when studying the disorder. We concentrated on females in this study as we wanted to avoid all of the confounders that a mixed population would present. The populations studied were representative of both non‐IBS adult females with low and moderate stress, on the one hand, and females with IBS, on the other. The IBS population contained IBS patients of all subtypes (constipation, diarrhea, mixed alternating), symptom severity (mild, moderate, severe), rates of prevalence of anxiety and depression (with a combined score of over 8), and impaired sleep quality (with a PSQI global score of over 5). The IBS cohort in this study was reflective of IBS patients in general where anxiety and/or stress have been reported to be present in up to 60% of all IBS suffers.^31^ PSS was not used as a screening entry criterion for the IBS patients, but we had seen a strong correlation between PSS and HADS anxiety, depression, and sleep quality in our healthy control population. This allowed us to focus on physiological measures. Psychological distress has been associated with more severe GI and non‐GI symptoms, fatigue, GI‐specific anxiety, and lower QOL.^11^, ^12^, ^13^, ^14^ The prevalence of depression in IBS patients has also been reported to be higher than in healthy controls (pooled difference 33% of the population). Importantly, reported levels of the HADS‐D levels of 5.4 were like those encountered in our patients.^32^

Evidence from clinical and experimental studies showed that psychological stresses have marked impacts on intestinal sensation, motility, secretion, and permeability; effects likely mediated through interactions between mucosal immune activation, alterations in central nervous system, peripheral neurons, and gastrointestinal microbiota^33^ Stress‐induced alterations in neuro‐endocrine‐immune pathways act on the gut–brain axis and microbiota–gut–brain axis, and, thereby, precipitate symptomatic exaggeration in IBS.^6^ The hypothalamic pituitary–adrenal (HPA) axis has been proposed as a mediator of brain–gut communication in IBS^34^, ^35^ with salivary cortisol providing an accessible measure of basal HPA‐axis activity.^36^ The stress response in IBS differs based upon sex, in that males with IBS exhibit an increased cortisol response after ACTH administration when compared with male healthy controls, whereas females with IBS exhibit a decreased response when compared with female healthy controls.^37^ The hippocampus, which exerts negative feedback on the HPA axis via glucocorticoid and mineralocorticoid receptors, shows sex differences in response to stressors, with less remodeling of hippocampal CA3 dendrites in females after chronic stress^38^ and in gene expression including in those related to glucocorticoid receptors.^39^ Based on our cortisol results, these studies suggest that stress is a major contributor to IBS symptoms in female patients.

In this study, we found that female IBS patients had higher baseline morning salivary cortisol levels than non‐IBS stressed controls in agreement with other studies^40^, ^41^ despite subjects with moderate stress and IBS demonstrating similar patterns of anxiety and depression. This contrasts with two other studies who have reported decreased baseline morning cortisol levels in patients with IBS.^42^, ^43^ However, subjects in the latter studies did not have elevated levels of depression or anxiety. In addition to the higher baseline morning cortisol level, in this study, we found that female IBS subjects had a blunted CAR compared to non‐IBS stress controls. This observation agrees with the study by Suarez and colleagues,^41^ who saw a similarly blunted CAR in female IBS patients with psychiatric co‐morbidities. Interestingly a blunted CAR was observed in young adult women with current mild‐to‐moderate clinical depression^44^ and those depressed subjects enrolled in psychotherapy.^45^ These studies suggest that patient sex and certain co‐morbidities contribute to the blunted CAR in IBS. Indeed, changes in CAR have been previously associated with anxiety, depression, and bad sleep quality,^46^, ^47^, ^48^ and previous work has found that functional pain symptoms are related to reduced cortisol secretion.^49^ Hellhammer and Wade have postulated a two‐stage model of disorders with a blunted cortisol response: first, chronic stress results in prolonged hypercortisolism that later develops into hypocortisolism. In such circumstances, the adrenal glands remain hypertrophic but secrete lower levels of cortisol.^50^ Surprisingly, in this study, we found a negative correlation between IBS severity and CAR response but no correlation between CAR and anxiety or depression levels suggesting the relationships between self‐reported measures and stress response are complex. Indeed, a recent study showed a complex interaction between symptom severity and neurophysiological measures in IBS.^51^ However, despite this growing evidence implicating HPA axis in IBS symptom pathogenesis, interventions to manage stress in IBS are poorly developed. In this study, we used the COMBO trial to examine how IBS, anxiety, depression, and stress response evolved over time to further define their relationships.

*Bifidobacterium longum* 35624 strain has previously been shown to reduce IBS symptoms^18^, ^52^ while *B. longum* 1714 reduced the perceived stress and cortisol output in response to an acute stressor.^19^ The combination of these strains resulted in a normalization of both the IBS symptoms and stress response in IBS patients. However, the effects on these parameters wane in the washout period. Psychological co‐morbidities are common in IBS patients and have been shown previously to be interwoven in the complex pathophysiological and clinical picture of IBS.^4^ In this study, symptom severity correlated with depression levels but not HADS anxiety or sleep quality. Interestingly, the combination of strains reduced anxiety and depression levels and improved sleep quality and visceral perception but, surprisingly, and in contrast to IBS symptoms and the stress response, once treatment stopped anxiety and depression levels continued to improve. We clarify that the normalization of CAR is transient and that the decrease in depression persists for 8 weeks. After this time, cortisol levels return to baseline levels. The time course of the IBS symptom response paralleled that of the cortisol response but not that of anxiety and depression. In agreement with our findings, a previous study showed that selective serotonin reuptake inhibitors (SSRI) may regulate the function of the HPA axis by reducing the baseline morning cortisol level and increasing the CAR.^53^ These effects correlated with therapeutic effects in the treatment of depression. Interestingly SSRIs have been reported to reduce global symptoms and abdominal pain in constipation predominant IBS patients^15^, ^17^ Hypocortisolism or a blunted CAR response is also present in chronic fatigue syndrome^54^ and potentially reversible by cognitive behavior therapy (CBT).^55^ CBT has also been shown to benefit IBS patients, perhaps due to its impact on the CAR response. Sustained stress exposure in IBS can result in HPA axis “fatigue” or blunting, glucocorticoid‐resistance, and an increase in systemic proinflammatory cytokines including TNF‐α contributing to the neuroinflammation manifested in depression.^56^, ^57^ One possible explanation could be that the probiotics are restoring HPA axis activity in the form of increased production of cortisol via the induction of a generalized compensatory anti‐inflammatory response. This could attenuate Th1 cell‐mediated immune responses and inhibit the synthesis of cytokines including TNFα, thereby, dampening neuroinflammation and reducing anxiety and depressive symptoms and improving sleep quality for up to 8 weeks post‐treatment. These findings suggest that the stress response is a major driver of symptoms in females with IBS and that beneficial clinical responses may have been achieved through a restoration of the stress response in these effects.^16^, ^58^

Very few studies have studied the effectiveness of probiotics on psychological symptoms in IBS patients. Treatment with the probiotic *B. longum* subsp. *longum* NCC3001 reduced depression scores and altered brain activity in patients with IBS in a placebo‐controlled pilot study.^26^ However, this study has been criticized for small numbers and differences at baseline in depression scores.^59^ Furthermore, a recent study showed that a combination of three *Bifidobacterium and Lactobacillus* strains improved VSI and IBS QOL.^60^ However, in contrast to our study, both studies showed no significant effect on bowel symptoms. Interestingly, in a 6‐week study, a probiotic mixture was found to lower depression, anxiety, and sleep subscale with the effects maintained after 3 weeks of washout.^61^ This suggests that, like our study, that psychological effects may feature a longer time course and associated washout.

IBS has many elements involved in its pathophysiology. In addition to gastrointestinal and depressive/anxious symptoms, underlying low‐grade inflammation is known to affect both symptomatologies.^1^, ^62^ In contrast to other studies,^63^, ^64^, ^65^ we did not see any significant difference in the plasma levels of TNF‐α between our asymptomatic non‐IBS controls and IBS subjects. Even though the levels of TNF‐α were not increased at baseline in the IBS patients, TNF‐α levels after treatment were significantly decreased over the treatment and washout periods, the time course of these effects paralleled depression levels but not IBS symptoms in response to the combination of strains. This agrees with previous studies which demonstrated the ability of *B. longum* 35624 to reduce inflammatory responses.^66^, ^67^ Furthermore, the reduction in peripheral proinflammatory cytokines was found to be related to a clinical improvement in HADS‐D of ≥2 and improvements in sleep quality, VSI, and anxiety levels. This agrees with a case–control trial, where the authors demonstrated that abnormal levels of cytokines, including TNF‐α, were significantly correlated with the severity of depressive and anxiety mood symptoms.^68^ This highlights the relationships between proinflammatory cytokines and depression/anxiety in IBS patients.^62^

In agreement with our observation that the CAR increased we also saw increases in the plasma levels of the neurotrophin BDNF after 4 and 12 weeks of treatment. This leads us to propose that an intriguing link between the effects on BDNF and the CAR may be due to improving hippocampal functional integrity and related to hippocampal neurogenesis and likely related to its positive effects on well‐being and depression. Interestingly, previous work has shown that hippocampal functioning promotes functioning of the CAR.^69^, ^70^ Specifically, patients with hippocampal damage have reduced CAR dynamics,^71^ and positive associations between hippocampal volume and CAR amplitude have been demonstrated.^72^ In addition, we speculate that the effects of on depression could relate to the decrease in the proinflammatory cytokines including TNF‐α. It has been shown that responders to the anti‐depressive medications agomelatine, fluoxetine, and mirtazapine in major depressive disorders exhibited both increased levels of circulating BDNF and decreases in circulating TNF‐α.^73^, ^74^ It is possible that changes in BDNF levels in depression might parallel a proinflammatory phenotype.^75^ This modulatory effect of neuroinflammation on neurotrophins could result from the integration of many mechanisms, including the hypothalamus pituitary axis and CAR.^76^

We acknowledge the limitations of the findings in our open‐label study which recruited subjects to study a subset of IBS patients who are comorbid with anxiety and depression. Usually, randomized double‐blind placebo‐controlled studies are state of the art in clinical medicine and IBS studies can involve a significant and variable placebo effect so these findings should be further explored in a placebo‐controlled study. However, using the open‐label study, we were able to provide valuable information on the efficacy and interaction of the two strains in combination, estimate the carryover effect, determine the washout period and provide reliable data on subject characteristics and variation in the parameters measured, as well as showing the therapeutic potential of this combination product in this defined population. We feel that this study provides important stand‐alone information on the longitudinal effects of these probiotics on both the central and visceral aspects of IBS in females, that will be useful to inform study designs for future trials in these areas.

In conclusion, by comparing baseline features in IBS with those of low and moderate non‐IBS stress controls and following the time course of the response to a probiotic combination with demonstrated central and peripheral effects, we were able to examine the relationships between symptoms, psychological co‐morbidities, and stress responses among females with IBS. Our findings suggest that these relationships are, indeed, complex and are influenced by symptom severity and predominant subtype. Furthermore, the time course of the response to the combination probiotic suggests that different mechanisms may underpin IBS symptoms, on the one hand, which appear linked to the stress response, and comorbid anxiety and depression and, on the other, seem to parallel circulating levels of the important proinflammatory cytokines such as TNFα. It is unclear what is the driver of symptoms; certainly, comorbid depression could play a central role in the patients studied here and our findings may not be applicable to IBS patients without comorbid anxiety and depression.

## AUTHOR CONTRIBUTIONS

DG, EM, and EQ conceived, planned, and oversaw the studies, and interpreted the data and wrote the paper. DG and HTTT performed laboratory experiments. DG, ION, ISL, and HTTT performed data analysis. All authors contributed to reviewing the paper and agreed the final version for submission.

## FUNDING INFORMATION

No funding declared

## DISCLOSURE

DG, EM, and HTTT are employees of PrecisionBiotics Group Limited which in part of Novozymes A/S Denmark. ISL is an employee of Novozymes A/S. Io'N has nothing to declare. EQ serves as a consultant to Precisionbiotics Group Limited.

## Supporting information


Figure S1

Figure S2

Figure S3

Figure S4

Figure S5

Table S1

Table S2

Table S3

Appendix S1

Appendix S2

## References

[1] Enck P , Aziz Q , Barbara G , et al. Irritable bowel syndrome. Nat Rev Dis Primers. 2016;2:16014. 27159638 10.1038/nrdp.2016.14 PMC5001845

[2] Sperber AD , Bangdiwala SI , Drossman DA , et al. Worldwide prevalence and burden of functional gastrointestinal disorders, results of Rome foundation global study. Gastroenterology. 2021;160(1):99‐114 e113. 32294476 10.1053/j.gastro.2020.04.014

[3] O'Malley D , Buckley MM , McKernan DP , Quigley EM , Cryan JF , Dinan TG . Soluble mediators in plasma from irritable bowel syndrome patients excite rat submucosal neurons. Brain Behav Immun. 2015;44:57‐67. 25150006 10.1016/j.bbi.2014.08.005

[4] Midenfjord I , Polster A , Sjovall H , Tornblom H , Simren M . Anxiety and depression in irritable bowel syndrome: exploring the interaction with other symptoms and pathophysiology using multivariate analyses. Neurogastroenterol Motil. 2019;31(8):e13619. 31056802 10.1111/nmo.13619

[5] Drossman DA . Functional gastrointestinal disorders: history, pathophysiology, clinical features and Rome IV. Gastroenterology. 2016. doi:10.1053/j.gastro.2016.02.032. Online ahead of print. 27144617

[6] O'Malley D , Quigley EM , Dinan TG , Cryan JF . Do interactions between stress and immune responses lead to symptom exacerbations in irritable bowel syndrome? Brain Behav Immun. 2011;25(7):1333‐1341. 21536124 10.1016/j.bbi.2011.04.009

[7] Zamani M , Alizadeh‐Tabari S , Zamani V . Systematic review with meta‐analysis: the prevalence of anxiety and depression in patients with irritable bowel syndrome. Aliment Pharmacol Ther. 2019;50(2):132‐143. 31157418 10.1111/apt.15325

[8] Hu Z , Li M , Yao L , et al. The level and prevalence of depression and anxiety among patients with different subtypes of irritable bowel syndrome: a network meta‐analysis. BMC Gastroenterol. 2021;21(1):23. 33413140 10.1186/s12876-020-01593-5 PMC7791666

[9] Posserud I , Agerforz P , Ekman R , Bjornsson ES , Abrahamsson H , Simren M . Altered visceral perceptual and neuroendocrine response in patients with irritable bowel syndrome during mental stress. Gut. 2004;53(8):1102‐1108. 15247175 10.1136/gut.2003.017962 PMC1774150

[10] Vu J , Kushnir V , Cassell B , Gyawali CP , Sayuk GS . The impact of psychiatric and extraintestinal comorbidity on quality of life and bowel symptom burden in functional GI disorders. Neurogastroenterol Motil. 2014;26(9):1323‐1332. 25070610 10.1111/nmo.12396

[11] Cho HS , Park JM , Lim CH , et al. Anxiety, depression and quality of life in patients with irritable bowel syndrome. Gut Liver. 2011;5(1):29‐36. 21461069 10.5009/gnl.2011.5.1.29 PMC3065090

[12] North CS , Downs D , Clouse RE , et al. The presentation of irritable bowel syndrome in the context of somatization disorder. Clin Gastroenterol Hepatol. 2004;2(9):787‐795. 15354279 10.1016/s1542-3565(04)00350-7

[13] Patel P , Bercik P , Morgan DG , et al. Irritable bowel syndrome is significantly associated with somatisation in 840 patients, which may drive bloating. Aliment Pharmacol Ther. 2015;41(5):449‐458. 25586008 10.1111/apt.13074

[14] Thijssen AY , Jonkers DM , Leue C , et al. Dysfunctional cognitions, anxiety and depression in irritable bowel syndrome. J Clin Gastroenterol. 2010;44(10):e236‐e241. 20733511 10.1097/MCG.0b013e3181eed5d8

[15] Xie C , Tang Y , Wang Y , et al. Efficacy and safety of antidepressants for the treatment of irritable bowel syndrome: a meta‐analysis. PLoS One. 2015;10(8):e0127815. 26252008 10.1371/journal.pone.0127815 PMC4529302

[16] Hauser G , Pletikosic S , Tkalcic M . Cognitive behavioral approach to understanding irritable bowel syndrome. World J Gastroenterol. 2014;20(22):6744‐6758. 24944466 10.3748/wjg.v20.i22.6744 PMC4051915

[17] Ford AC , Lacy BE , Harris LA , Quigley EMM , Moayyedi P . Effect of antidepressants and psychological therapies in irritable bowel syndrome: an updated systematic review and meta‐analysis. Am J Gastroenterol. 2019;114(1):21‐39. 30177784 10.1038/s41395-018-0222-5

[18] Whorwell PJ , Altringer L , Morel J , et al. Efficacy of an encapsulated probiotic *Bifidobacterium infantis* 35624 in women with irritable bowel syndrome. Am J Gastroenterol. 2006;101(7):1581‐1590. 16863564 10.1111/j.1572-0241.2006.00734.x

[19] Allen AP , Hutch W , Borre YE , et al. *Bifidobacterium longum* 1714 as a translational psychobiotic: modulation of stress, electrophysiology and neurocognition in healthy volunteers. Transl Psychiatry. 2016;6(11):e939. 27801892 10.1038/tp.2016.191 PMC5314114

[20] Cohen S , Kamarck T , Mermelstein R . A global measure of perceived stress. J Health Soc Behav. 1983;24(4):385‐396. 6668417

[21] Francis CY , Morris J , Whorwell PJ . The irritable bowel severity scoring system: a simple method of monitoring irritable bowel syndrome and its progress. Aliment Pharmacol Ther. 1997;11(2):395‐402. 9146781 10.1046/j.1365-2036.1997.142318000.x

[22] Zigmond AS , Snaith RP . The hospital anxiety and depression scale. Acta Psychiatr Scand. 1983;67(6):361‐370. 6880820 10.1111/j.1600-0447.1983.tb09716.x

[23] Herrmann C . International experiences with the hospital anxiety and depression scale—a review of validation data and clinical results. J Psychosom Res. 1997;42(1):17‐41. 9055211 10.1016/s0022-3999(96)00216-4

[24] Bjelland I , Dahl AA , Haug TT , Neckelmann D . The validity of the hospital anxiety and depression scale. An updated literature review. J Psychosom Res. 2002;52(2):69‐77. 11832252 10.1016/s0022-3999(01)00296-3

[25] Puhan MA , Frey M , Buchi S , Schunemann HJ . The minimal important difference of the hospital anxiety and depression scale in patients with chronic obstructive pulmonary disease. Health Qual Life Outcomes. 2008;6:46. 18597689 10.1186/1477-7525-6-46 PMC2459149

[26] Pinto‐Sanchez MI , Hall GB , Ghajar K , et al. Probiotic *Bifidobacterium longum* NCC3001 reduces depression scores and alters brain activity: a pilot study in patients with irritable bowel syndrome. Gastroenterology. 2017;153(2):448‐459 e448. 28483500 10.1053/j.gastro.2017.05.003

[27] Labus JS , Bolus R , Chang L , et al. The visceral sensitivity index: development and validation of a gastrointestinal symptom‐specific anxiety scale. Aliment Pharmacol Ther. 2004;20(1):89‐97. 10.1111/j.1365-2036.2004.02007.x 15225175

[28] Buysse DJ , Reynolds CF III , Monk TH , Berman SR , Kupfer DJ . The Pittsburgh sleep quality index: a new instrument for psychiatric practice and research. Psychiatry Res. 1989;28(2):193‐213. 2748771 10.1016/0165-1781(89)90047-4

[29] Pruessner JC , Kirschbaum C , Meinlschmid G , Hellhammer DH . Two formulas for computation of the area under the curve represent measures of total hormone concentration versus time‐dependent change. Psychoneuroendocrinology. 2003;28(7):916‐931. 12892658 10.1016/s0306-4530(02)00108-7

[30] Josse J , Chavent M , Liquet B , Husson F . Handling missing values with regularized iterative multiple correspondence analysis. Journal of Classification. 2012;29(1):91‐116.

[31] Hartono JL , Mahadeva S , Goh KL . Anxiety and depression in various functional gastrointestinal disorders: do differences exist? J Dig Dis. 2012;13(5):252‐257. 22500787 10.1111/j.1751-2980.2012.00581.x

[32] Shah E , Rezaie A , Riddle M , Pimentel M . Psychological disorders in gastrointestinal disease: epiphenomenon, cause or consequence? Ann Gastroenterol. 2014;27(3):224‐230. 24974805 PMC4073018

[33] Qin HY , Cheng CW , Tang XD , Bian ZX . Impact of psychological stress on irritable bowel syndrome. World J Gastroenterol. 2014;20(39):14126‐14131. 25339801 10.3748/wjg.v20.i39.14126 PMC4202343

[34] Mayer EA , Naliboff BD , Chang L , Coutinho SV . Stress and irritable bowel syndrome. Am J Physiol Gastrointest Liver Physiol. 2001;280(4):G519‐G524. 11254476 10.1152/ajpgi.2001.280.4.G519

[35] Manabe N , Tanaka T , Hata J , Kusunoki H , Haruma K . Pathophysiology underlying irritable bowel syndrome‐‐from the viewpoint of dysfunction of autonomic nervous system activity. J Smooth Muscle Res. 2009;45(1):15‐23. 19377269 10.1540/jsmr.45.15

[36] Holleman M , Vreeburg SA , Dekker JJ , Penninx BW . The relationships of working conditions, recent stressors and childhood trauma with salivary cortisol levels. Psychoneuroendocrinology. 2012;37(6):801‐809. 22000684 10.1016/j.psyneuen.2011.09.012

[37] Videlock EJ , Shih W , Adeyemo M , et al. The effect of sex and irritable bowel syndrome on HPA axis response and peripheral glucocorticoid receptor expression. Psychoneuroendocrinology. 2016;69:67‐76. 27038676 10.1016/j.psyneuen.2016.03.016 PMC4977028

[38] McEwen BS , Nasca C , Gray JD . Stress effects on neuronal structure: hippocampus, amygdala, and prefrontal cortex. Neuropsychopharmacology. 2016;41(1):3‐23. 26076834 10.1038/npp.2015.171 PMC4677120

[39] Quinn MA , Cidlowski JA . Endogenous hepatic glucocorticoid receptor signaling coordinates sex‐biased inflammatory gene expression. FASEB J. 2016;30(2):971‐982. 26581598 10.1096/fj.15-278309 PMC4714552

[40] Patacchioli FR , Angelucci L , Dellerba G , Monnazzi P , Leri O . Actual stress, psychopathology and salivary cortisol levels in the irritable bowel syndrome (IBS). J Endocrinol Invest. 2001;24(3):173‐177. 11314746 10.1007/BF03343838

[41] Suarez‐Hitz KA , Otto B , Bidlingmaier M , Schwizer W , Fried M , Ehlert U . Altered psychobiological responsiveness in women with irritable bowel syndrome. Psychosom Med. 2012;74(2):221‐231. 22286854 10.1097/PSY.0b013e318244fb82

[42] Bohmelt AH , Nater UM , Franke S , Hellhammer DH , Ehlert U . Basal and stimulated hypothalamic‐pituitary‐adrenal axis activity in patients with functional gastrointestinal disorders and healthy controls. Psychosom Med. 2005;67(2):288‐294. 15784796 10.1097/01.psy.0000157064.72831.ba

[43] Kennedy PJ , Cryan JF , Quigley EM , Dinan TG , Clarke G . A sustained hypothalamic‐pituitary‐adrenal axis response to acute psychosocial stress in irritable bowel syndrome. Psychol Med. 2014;44(14):3123‐3134. 25065954 10.1017/S003329171400052X

[44] Stetler C , Miller GE . Blunted cortisol response to awakening in mild to moderate depression: regulatory influences of sleep patterns and social contacts. J Abnorm Psychol. 2005;114(4):697‐705. 16351390 10.1037/0021-843X.114.4.697

[45] Huber TJ , Issa K , Schik G , Wolf OT . The cortisol awakening response is blunted in psychotherapy inpatients suffering from depression. Psychoneuroendocrinology. 2006;31(7):900‐904. 16707227 10.1016/j.psyneuen.2006.03.005

[46] Dedovic K , Ngiam J . The cortisol awakening response and major depression: examining the evidence. Neuropsychiatr Dis Treat. 2015;11:1181‐1189. 25999722 10.2147/NDT.S62289 PMC4437603

[47] Hek K , Direk N , Newson RS , et al. Anxiety disorders and salivary cortisol levels in older adults: a population‐based study. Psychoneuroendocrinology. 2013;38(2):300‐305. 22776419 10.1016/j.psyneuen.2012.06.006

[48] Fekedulegn D , Innes K , Andrew ME , et al. Sleep quality and the cortisol awakening response (CAR) among law enforcement officers: the moderating role of leisure time physical activity. Psychoneuroendocrinology. 2018;95:158‐169. 29864672 10.1016/j.psyneuen.2018.05.034 PMC6401560

[49] Ehlert U , Nater UM , Bohmelt A . High and low unstimulated salivary cortisol levels correspond to different symptoms of functional gastrointestinal disorders. J Psychosom Res. 2005;59(1):7‐10. 16126090 10.1016/j.jpsychores.2005.03.005

[50] Hellhammer DH , Wade S . Endocrine correlates of stress vulnerability. Psychother Psychosom. 1993;60(1):8‐17. 8234641 10.1159/000288675

[51] Midenfjord I , Polster A , Sjovall H , Friberg P , Tornblom H , Simren M . Associations among neurophysiology measures in irritable bowel syndrome (IBS) and their relevance for IBS symptoms. Sci Rep. 2020;10(1):9794. 32555219 10.1038/s41598-020-66558-w PMC7300023

[52] O'Mahony L , McCarthy J , Kelly P , et al. Lactobacillus and bifidobacterium in irritable bowel syndrome: symptom responses and relationship to cytokine profiles. Gastroenterology. 2005;128(3):541‐551. 15765388 10.1053/j.gastro.2004.11.050

[53] Ruhe HG , Khoenkhoen SJ , Ottenhof KW , Koeter MW , Mocking RJ , Schene AH . Longitudinal effects of the SSRI paroxetine on salivary cortisol in major depressive disorder. Psychoneuroendocrinology. 2015;52:261‐271. 25544738 10.1016/j.psyneuen.2014.10.024

[54] Nijhof SL , Rutten JM , Uiterwaal CS , Bleijenberg G , Kimpen JL , Putte EM . The role of hypocortisolism in chronic fatigue syndrome. Psychoneuroendocrinology. 2014;42:199‐206. 24636516 10.1016/j.psyneuen.2014.01.017

[55] Roberts AD , Papadopoulos AS , Wessely S , Chalder T , Cleare AJ . Salivary cortisol output before and after cognitive behavioural therapy for chronic fatigue syndrome. J Affect Disord. 2009;115(1–2):280‐286. 18937978 10.1016/j.jad.2008.09.013

[56] Webster JI , Tonelli L , Sternberg EM . Neuroendocrine regulation of immunity. Annu Rev Immunol. 2002;20:125‐163. 11861600 10.1146/annurev.immunol.20.082401.104914

[57] Dantzer R , Kelley KW . Twenty years of research on cytokine‐induced sickness behavior. Brain Behav Immun. 2007;21(2):153‐160. 17088043 10.1016/j.bbi.2006.09.006 PMC1850954

[58] Everitt HA , Landau S , O'Reilly G , et al. Cognitive behavioural therapy for irritable bowel syndrome: 24‐month follow‐up of participants in the ACTIB randomised trial. Lancet Gastroenterol Hepatol. 2019;4(11):863‐872. 31492643 10.1016/S2468-1253(19)30243-2 PMC7026694

[59] Meyer C , Vassar M . The fragility of probiotic *Bifidobacterium longum* NCC3001 use for depression in patients with irritable bowel syndrome. Gastroenterology. 2018;154(3):764. 10.1053/j.gastro.2017.09.055 29352957

[60] Lorenzo‐Zuniga V , Llop E , Suarez C , et al. I.31, a new combination of probiotics, improves irritable bowel syndrome‐related quality of life. World J Gastroenterol. 2014;20(26):8709‐8716. 25024629 10.3748/wjg.v20.i26.8709 PMC4093724

[61] Marotta A , Sarno E , Del Casale A , et al. Effects of probiotics on cognitive reactivity, mood, and sleep quality. Front Psych. 2019;10:164. 10.3389/fpsyt.2019.00164 PMC6445894 30971965

[62] Liebregts T , Adam B , Bredack C , et al. Immune activation in patients with irritable bowel syndrome. Gastroenterology. 2007;132(3):913‐920. 17383420 10.1053/j.gastro.2007.01.046

[63] Rana SV , Sharma S , Sinha SK , Parsad KK , Malik A , Singh K . Pro‐inflammatory and anti‐inflammatory cytokine response in diarrhoea‐predominant irritable bowel syndrome patients. Trop Gastroenterol. 2012;33(4):251‐256. 23923350 10.7869/tg.2012.66

[64] Seyedmirzaee S , Hayatbakhsh MM , Ahmadi B , et al. Serum immune biomarkers in irritable bowel syndrome. Clin Res Hepatol Gastroenterol. 2016;40(5):631‐637. 26850360 10.1016/j.clinre.2015.12.013

[65] Schmulson M . Irritable bowel syndrome (IBS) in the 2012 DDW. Rev Gastroenterol Mex. 2012;77(Suppl. 1):50‐52. 22939481 10.1016/j.rgmx.2012.07.020

[66] Groeger D , O'Mahony L , Murphy EF , et al. *Bifidobacterium infantis* 35624 modulates host inflammatory processes beyond the gut. Gut Microbes. 2013;4(4):325‐339. 23842110 10.4161/gmic.25487 PMC3744517

[67] Konieczna P , Groeger D , Ziegler M , et al. *Bifidobacterium infantis* 35624 administration induces Foxp3 T regulatory cells in human peripheral blood: potential role for myeloid and plasmacytoid dendritic cells. Gut. 2012;61(3):354‐366. 22052061 10.1136/gutjnl-2011-300936

[68] Zhen Y , Chu C , Zhou S , Qi M , Shu R . Imbalance of tumor necrosis factor‐alpha, interleukin‐8 and interleukin‐10 production evokes barrier dysfunction, severe abdominal symptoms and psychological disorders in patients with irritable bowel syndrome‐associated diarrhea. Mol Med Rep. 2015;12(4):5239‐5245. 26180016 10.3892/mmr.2015.4079

[69] Fries E , Dettenborn L , Kirschbaum C . The cortisol awakening response (CAR): facts and future directions. Int J Psychophysiol. 2009;72(1):67‐73. 18854200 10.1016/j.ijpsycho.2008.03.014

[70] Clow A , Hucklebridge F , Stalder T , Evans P , Thorn L . The cortisol awakening response: more than a measure of HPA axis function. Neurosci Biobehav Rev. 2010;35(1):97‐103. 20026350 10.1016/j.neubiorev.2009.12.011

[71] Buchanan TW , Kern S , Allen JS , Tranel D , Kirschbaum C . Circadian regulation of cortisol after hippocampal damage in humans. Biol Psychiatry. 2004;56(9):651‐656. 15522248 10.1016/j.biopsych.2004.08.014

[72] Pruessner M , Pruessner JC , Hellhammer DH , Bruce Pike G , Lupien SJ . The associations among hippocampal volume, cortisol reactivity, and memory performance in healthy young men. Psychiatry Res. 2007;155(1):1‐10. 17395434 10.1016/j.pscychresns.2006.12.007

[73] Gupta K , Gupta R , Bhatia MS , Tripathi AK , Gupta LK . Effect of agomelatine and fluoxetine on HAM‐D score, serum brain‐derived neurotrophic factor, and tumor necrosis factor‐alpha level in patients with major depressive disorder with severe depression. J Clin Pharmacol. 2017;57(12):1519‐1526. 28833192 10.1002/jcph.963

[74] Gupta R , Gupta K , Tripathi AK , Bhatia MS , Gupta LK . Effect of mirtazapine treatment on serum levels of brain‐derived neurotrophic factor and tumor necrosis factor‐alpha in patients of major depressive disorder with severe depression. Pharmacology. 2016;97(3–4):184‐188. 26854819 10.1159/000444220

[75] Calabrese F , Rossetti AC , Racagni G , Gass P , Riva MA , Molteni R . Brain‐derived neurotrophic factor: a bridge between inflammation and neuroplasticity. Front Cell Neurosci. 2014;8:430. 25565964 10.3389/fncel.2014.00430 PMC4273623

[76] Rivest S . Interactions between the immune and neuroendocrine systems. Prog Brain Res. 2010;181:43‐53. 20478432 10.1016/S0079-6123(08)81004-7

