# Supplementary figures and images for: Interactions between symptoms and psychological status in irritable bowel syndrome: An exploratory study of the impact of a probiotic combination
Source: Neurogastroenterol Motil. 2022 Sep 30;35(1):e14477. doi: 10.1111/nmo.14477 (PMC10078522; doi:10.1111/nmo.14477)

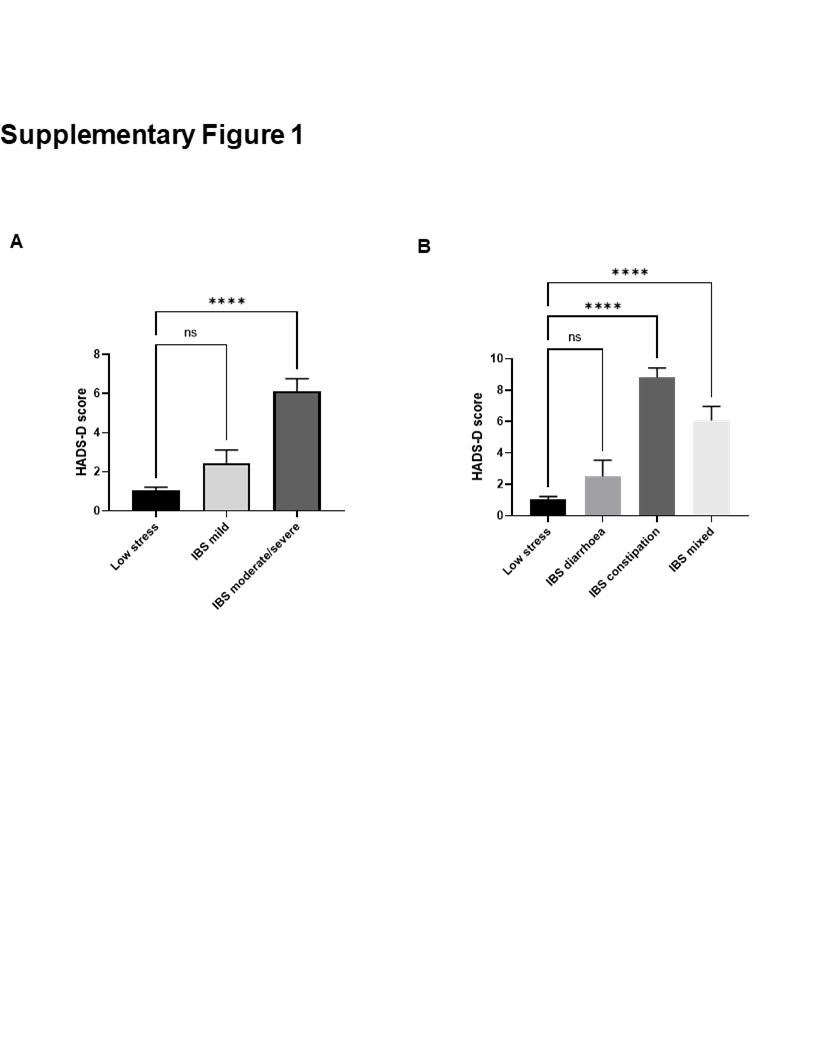

Supplement: Supplementary file 1 — Figure S1 [file NMO-35-0-s009.tif]

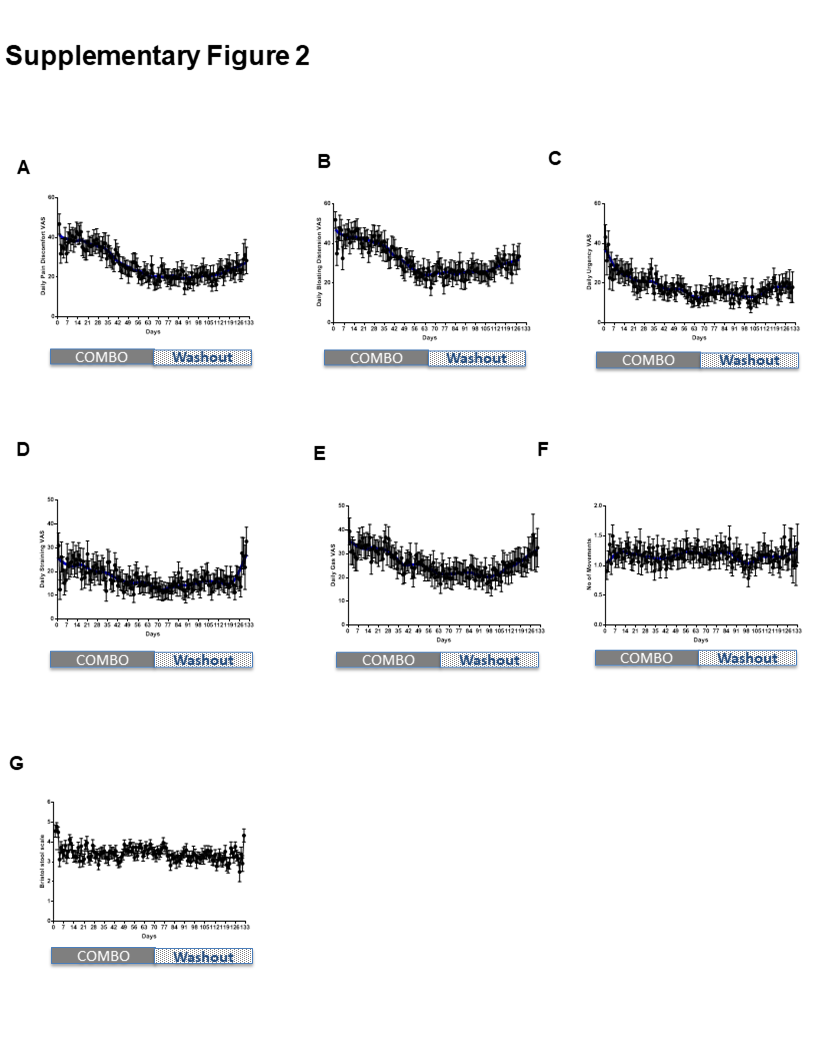

Supplement: Supplementary file 2 — Figure S2 [file NMO-35-0-s005.tif]

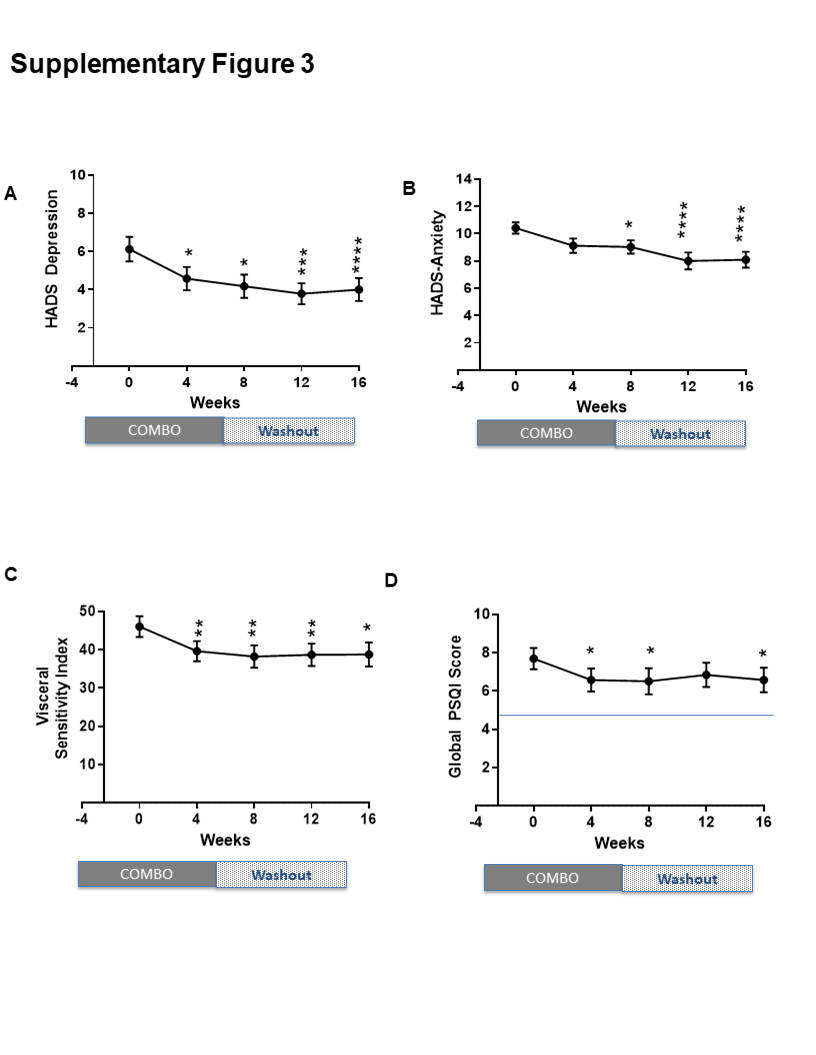

Supplement: Supplementary file 3 — Figure S3 [file NMO-35-0-s004.tif]

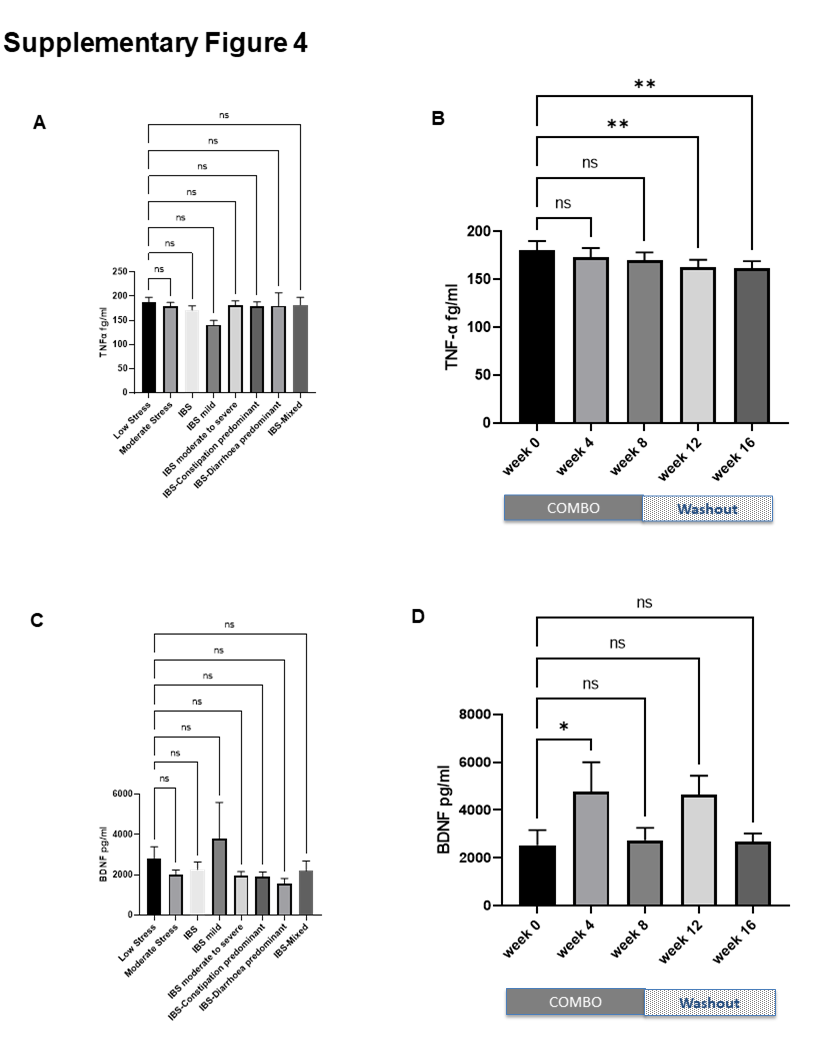

Supplement: Supplementary file 4 — Figure S4 [file NMO-35-0-s007.tif]

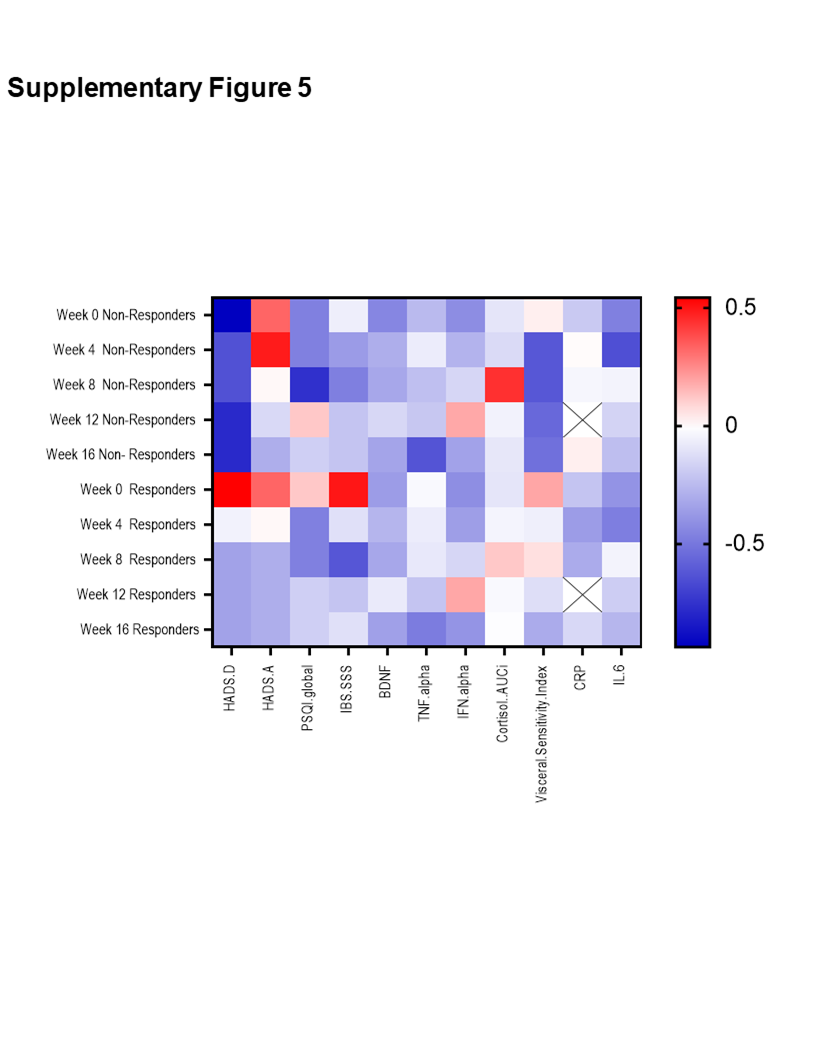

Supplement: Supplementary file 5 — Figure S5 [file NMO-35-0-s002.tif]
