# Supplementary material for: Interactions between symptoms and psychological status in irritable bowel syndrome: An exploratory study of the impact of a probiotic combination
Source: Neurogastroenterol Motil. 2022 Sep 30;35(1):e14477. doi: 10.1111/nmo.14477 (PMC10078522; doi:10.1111/nmo.14477)
Supplement: Supplementary file 6 — Table S1 [file NMO-35-0-s010.docx]

**Supplementary Table 1**

|  | **Median (IQR)** | | |
| --- | --- | --- | --- |
| **Plasma biomarkers** | **Low**  **Stress** | **Moderate stress** | **IBS** |
| IL-6 fg/ml | 1206(855.8-1727 | 1001(6941-1381) | 807.0(615.7-1180 |
| CRP mg/L | 0.5982 (0.3960-1.258) | 2.566 (1.035-3.846) | 1.726 (0.8869-4.350) |
| TNF-α  fg/ml | 194.8 (148.3-229.6) | 172.0 (149.4-212.5) | 165.8 (139.1-191.1) |
| IFN-α  fg/ml | 89.21 (52.49-167.2) | 118.8 (61.88-) | 76.45 (57.81-114.0) |
| BDNF pg/ml | 2950 (1773-15688) | 2479 (1739-4714) | 2657  (1689-14174) |
