# Supplementary material for: Interactions between symptoms and psychological status in irritable bowel syndrome: An exploratory study of the impact of a probiotic combination
Source: Neurogastroenterol Motil. 2022 Sep 30;35(1):e14477. doi: 10.1111/nmo.14477 (PMC10078522; doi:10.1111/nmo.14477)
Supplement: Supplementary file 7 — Table S2 [file NMO-35-0-s006.docx]

**Supplementary Table 2**

|  | **Change in IBS-SSS ≥ 50** | **Change in HADS-depression ≥ 2** | **Change in HADS-anxiety ≥ 2** | **PSQI global score Change in sleep category**  **(Poor to good)** |
| --- | --- | --- | --- | --- |
| Baseline | - | - | - | - |
| ∆ wk 4 | 66% | 52% | 36% | 21% |
| ∆ wk 8 | 82% | 48% | 45% | 30% |
| ∆ wk 12 | 55% | 55% | 61% | 27% |
| ∆ wk 16 | 40% | 61% | 61% | 27% |
