# Supplementary material for: Interactions between symptoms and psychological status in irritable bowel syndrome: An exploratory study of the impact of a probiotic combination
Source: Neurogastroenterol Motil. 2022 Sep 30;35(1):e14477. doi: 10.1111/nmo.14477 (PMC10078522; doi:10.1111/nmo.14477)
Supplement: Supplementary file 8 — Table S3 [file NMO-35-0-s001.docx]

**Supplementary Table 3**

| **ANCOVA comparison of responder to non-responder** | | | | |  |
| --- | --- | --- | --- | --- | --- |
|  | ∆ wk 4 | ∆ wk 8 | ∆ wk 12 | ∆ wk 16 |  |
| HADS- depression | P=0.0025 | P=0.0016 | P=0.0006 | P=0.0034 |  |
| HADS -Anxiety | P=0.0058 | NS | NS | NS |  |
| IBS-SSS | NS | NS | NS | NS |  |
| PSQI global  score | P=0.0720 | NS | P=0.0093 | P=0.0149 |  |
| Visceral Sensitivity Index | NS | NS | NS | NS |  |
| BDNF pg/ml | NS | NS | NS | NS |  |
| TNF-α fg/ml | NS | P=0.036 | NS | NS |  |
| IFN-α fg/ml | NS | NS | NS | NS |  |
| IL-6 fg/ml | NS | NS | P=0.045 | NS |  |
| CRP mg/L | P=0.0016 | P=0.010 | NS | P=0.013436 |  |
