# Supplementary material for: Interactions between symptoms and psychological status in irritable bowel syndrome: An exploratory study of the impact of a probiotic combination
Source: Neurogastroenterol Motil. 2022 Sep 30;35(1):e14477. doi: 10.1111/nmo.14477 (PMC10078522; doi:10.1111/nmo.14477)
Supplement: Supplementary file 9 — Appendix S1 [file NMO-35-0-s003.docx]

Supplementary Figure 1 *Psychometric Baseline Characteristics*

HADS Depression score was determined at baseline. Data shown are mean ± SEM. ****p < 0.0001 (1-way ANOVA comparison IBS mild, IBS moderate to severe versus low stress group). **(b)** HADS Depression score was determined at baseline. Data shown are mean ± SEM. ****p < 0.0001 (1-way ANOVA comparison IBS moderate to severe patients’ subtypes (diarrhoea, constipation and mixed) versus low stress group).

Supplementary Table 1 *Baseline Plasma biomarker levels*

Supplementary Figure 2. *Daily GI symptoms are improved in moderate to severe IBS patients before and after treatment with the COMBO*

The impact of the COMBO treatment on measures of **(a)** daily pain discomfort **(b)** daily bowel distension **(c)** daily urgency **(d)** daily straining **(e)** daily gas (f) number of bowel movements per day (g) Bristol scale stool for the type of stool. All data were collected using an E-diary every day for 16 weeks. Results are presented as a mean-SE

Supplementary Table 2. *Clinically relevant % changes before and after COMBO*

Supplementary Figure 3. *Psychometric symptoms are decreased in moderate to severe IBS patients before and after treatment with the COMBO*

(a) HADS depression and (b) HADS anxiety scores (c) Visceral sensitivity index (d) PSQI global score (e). Data shown are mean ± SEM. *p < 0.05, *p < 0.01, ****p < 0.0001 (1-way ANOVA comparison of week 0 to the other time points week 4, 8,12, and 16 patients before and after COMBO treatment.

Supplementary Table 3. *ANCOVA Analysis of HADS Depression improvements ≥2 Responders versus non responders*

Supplementary Figure 4. *Pro-inflammatory cytokine TNF-α are decreased and neurotrophic factor BDNF in moderate to severe IBS patients before and after treatment with the COMBO*

**(a)** Plasma TNF-α at baseline comparison of low stress to moderate stress and mild to moderate severe IBS. Data shown are mean ± SEM) **(b)** Plasma TNF-α before and after treatment with the COMBO. Data shown are mean ± SEM. *p < 0.05, *p < 0.01, ****p < 0.0001 (1-way ANOVA comparison of week 0 to the other time points week 4, 8,12, and 16 patients before and after COMBO treatment. **(c)** Plasma BDNF at baseline comparison of low stress to moderate stress and mild to moderate severe IBS. Data shown are mean ± SEM) **(d)** Plasma BDNF before and after treatment with the COMBO. Data shown are mean ± SEM. *p < 0.05, *p < 0.01, ****p < 0.0001 (1-way ANOVA comparison of week 0 to the other time points week 4, 8,12, and 16 patients before and after COMBO treatment.

Supplementary Figure 5. *Improvements in HADS Depression is a determining factor in selecting Responders and non-responders to the COMBO*

Heatmap showing individual mean biological endpoints and subjective questionnaires values from those had an improvement ≥2 in HADS depression and those who did not (ANCOVA comparison IBS responders vs. non-responders)
