# Supplementary material for: Interactions between symptoms and psychological status in irritable bowel syndrome: An exploratory study of the impact of a probiotic combination
Source: Neurogastroenterol Motil. 2022 Sep 30;35(1):e14477. doi: 10.1111/nmo.14477 (PMC10078522; doi:10.1111/nmo.14477)
Supplement: Supplementary file 10 — Appendix S2 [file NMO-35-0-s008.pdf]

The probiotic combination restored the blunted cortisol response associated with IBS which was mirrored by clinically significant improvements in overall IBS symptoms, but the effect lasted only for the duration of treatment. The probiotic combination reduced depression, anxiety scores and circulating levels of TNFα, as well as improving sleep quality.
